# Tumor suppressor menin is required for subunit-specific nAChR α5 transcription and nAChR-dependent presynaptic facilitation in cultured mouse hippocampal neurons

**DOI:** 10.1038/s41598-017-01825-x

**Published:** 2017-05-11

**Authors:** Angela M. Getz, Fenglian Xu, Frank Visser, Roger Persson, Naweed I. Syed

**Affiliations:** 1 Department of Cell Biology & Anatomy, Hotchkiss Brain Institute and Alberta Children’s Hospital Research Institute, University of Calgary, Calgary, Alberta T2N 1N4 Canada; 2 Department of Neuroscience, Hotchkiss Brain Institute, University of Calgary, Calgary, Alberta T2N 1N4 Canada; 3 Department of Physiology & Pharmacology, Hotchkiss Brain Institute, University of Calgary, Calgary, Alberta T2N 1N4 Canada; 4 Department of Biology, Saint Louis University, Saint Louis, Missouri 63103 USA; 5 BioAxial, Paris, 75014 France

## Abstract

In the central nervous system (CNS), cholinergic transmission induces synaptic plasticity that is required for learning and memory. However, our understanding of the development and maintenance of cholinergic circuits is limited, as the factors regulating the expression and clustering of neuronal nicotinic acetylcholine receptors (nAChRs) remain poorly defined. Recent studies from our group have implicated calpain-dependent proteolytic fragments of menin, the product of the *MEN1* tumor suppressor gene, in coordinating the transcription and synaptic clustering of nAChRs in invertebrate central neurons. Here, we sought to determine whether an analogous cholinergic mechanism underlies menin’s synaptogenic function in the vertebrate CNS. Our data from mouse primary hippocampal cultures demonstrate that menin and its calpain-dependent C-terminal fragment (C-menin) regulate the subunit-specific transcription and synaptic clustering of neuronal nAChRs, respectively. *MEN1* knockdown decreased nAChR α5 subunit expression, the clustering of α7 subunit-containing nAChRs at glutamatergic presynaptic terminals, and nicotine-induced presynaptic facilitation. Moreover, the number and function of glutamatergic synapses was unaffected by *MEN1* knockdown, indicating that the synaptogenic actions of menin are specific to cholinergic regulation. Taken together, our results suggest that the influence of menin on synapse formation and synaptic plasticity occur via modulation of nAChR channel subunit composition and functional clustering.

## Introduction

Synapse formation and synaptic plasticity in the central nervous system (CNS) require the coordination of nuclear transcription and site-specific targeting of nascent synaptic proteins in response to extracellular (e.g. neurotrophic factors^1^) and cell-cell (e.g. neuroligin-neurexin^2, 3^) signaling interactions. Mounting evidence from our group^4–6^ and others^7–9^ supports the conserved role of menin, the protein product of the *MEN1* (multiple endocrine neoplasia type 1) tumor suppressor gene^10^, in mediating synapse formation and synaptic plasticity in the CNS. Studies on the role of menin as a tumor suppressor indicate that it is a multifunction scaffold protein that integrates extracellular and cell-cell signaling interactions, intracellular molecular cascades, and nuclear transcription^11^. The molecular mechanisms underlying the synaptogenic function of menin, however, have not been well characterized. One particularly enigmatic aspect of menin is that whereas its expression is ubiquitous, it acts as a tumor suppressor only in certain cell types^12^. Considering that *MEN1* expression is found in most tissues, it is likely that as-yet uncharacterized molecular actions of menin may be necessary for specialized cellular functions that extend beyond its basic biological roles in genome maintenance^13^ and cell cycle regulation^14^. We have recently reported that the menin orthologue from the invertebrate mollusk *Lymnaea stagnalis* (*L*-*MEN1*/*L*-menin) induces the subunit-specific transcriptional upregulation and synaptic clustering of neuronal nicotinic acetylcholine receptors (*L*-nAChRs) during cholinergic synaptogenesis^6^. This work posed two important questions regarding menin’s neuronal molecular functions that remained unanswered: (i) does an analogous mechanism of action underlie menin’s synaptogenic function in the vertebrate CNS; and (ii) does menin act ubiquitously as a synaptogenic factor, or are its molecular actions specific to cholinergic synaptogenesis via the regulation of neuronal nAChRs?

In the mammalian CNS, the activation of cholinergic synapses induces synaptic modulation and plasticity events which are critically required for learning and memory^15, 16^. For instance, disruptions of cholinergic projections, their synaptic connections, and nAChR expression and function are early events central to the cognitive decline that occurs in neurodegenerative Alzheimer’s disease (AD)^17, 18^. Despite the importance of cholinergic synaptic activity to cognition, our understanding of the mechanisms governing the assembly, function and maintenance of central cholinergic synapses is incomplete. Specifically, dissection of the molecular factors regulating cholinergic circuits in the brain is hindered by the fact that (i) neuronal nAChRs are highly heterogeneous and channel subunit composition can be ambiguous, (ii) the mechanisms underlying the expression and subcellular targeting of distinct neuronal nAChR subunits or channel subtypes are not well understood, and (iii) the molecular scaffolds and/or adaptor molecules underlying the synaptic clustering of neuronal nAChRs are unidentified^19, 20^. The molecular determinants of cholinergic synaptogenesis at the neuromuscular junction (NMJ), agrin-MuSK-rapsyn and neuregulin-ErbB^21^, are expressed in central neurons and have been shown to modulate the formation and function of central synapses^22, 23^ as well as the expression and synaptic targeting of neuronal nAChRs^24–26^. However, rapsyn, the molecular scaffold for nAChR clustering in muscle cells^27^, is absent from synaptic clusters of nAChRs in neurons^28^. It therefore follows that central neurons employ an as-yet unidentified postsynaptic scaffold to cluster nAChRs. Considering our previous observations in *Lymnaea* that the synaptogenic effects of *L*-menin are mediated, in part, by the synaptic clustering of *L*-nAChRs via a calpain-dependent C-terminal proteolytic fragment (C-menin)^6^, there exists an intriguing possibility that C-menin may be a candidate molecular scaffold or adaptor for neuronal nAChRs. The mechanisms underlying the synaptogenic function of menin in the vertebrate CNS, however, have not yet been defined. As the highest levels of *MEN1* expression in the brain are found in the hippocampus^29^, a center for learning and memory which receives extensive cholinergic innervation and exhibits abundant nAChR expression^30^, it may be that one of the specialized cellular functions of menin is the regulation of nAChR expression and clustering in neurons.

In the present study, we employed mouse brain tissue and primary hippocampal cultures to explore the molecular mechanisms underlying the synaptogenic effects of menin. Here, we report that menin exhibits calpain-dependent proteolytic cleavage and differential subcellular distribution of the resulting fragments. Whereas full length menin localized to the nucleus, the N-terminal proteolytic fragment was cytoplasmically localized and the C-terminal proteolytic fragment was synaptically localized. The C-terminal menin fragment colocalized extensively with α7 subunit-containing nAChRs, and these co-clusters were specifically enhanced at glutamatergic presynaptic terminals. *MEN1* knockdown *in vitro* selectively inhibited transcription of the nAChR α5 subunit, but otherwise induced a general transcriptional upregulation. *MEN1* knockdown also reduced the clustering of α7 subunit-containing nAChRs at glutamatergic presynaptic terminals, and nicotine-induced presynaptic facilitation. However, the number and function of glutamatergic synapses was otherwise unaffected. Taken together, our results suggest that menin does not act as a ubiquitous synaptogenic factor, but that it is a specific regulator of neuronal nAChR subunit composition and functional clustering.

## Results

### Calpain-dependent proteolytic fragments of menin are differentially localized within neurons

**Figure 1:**
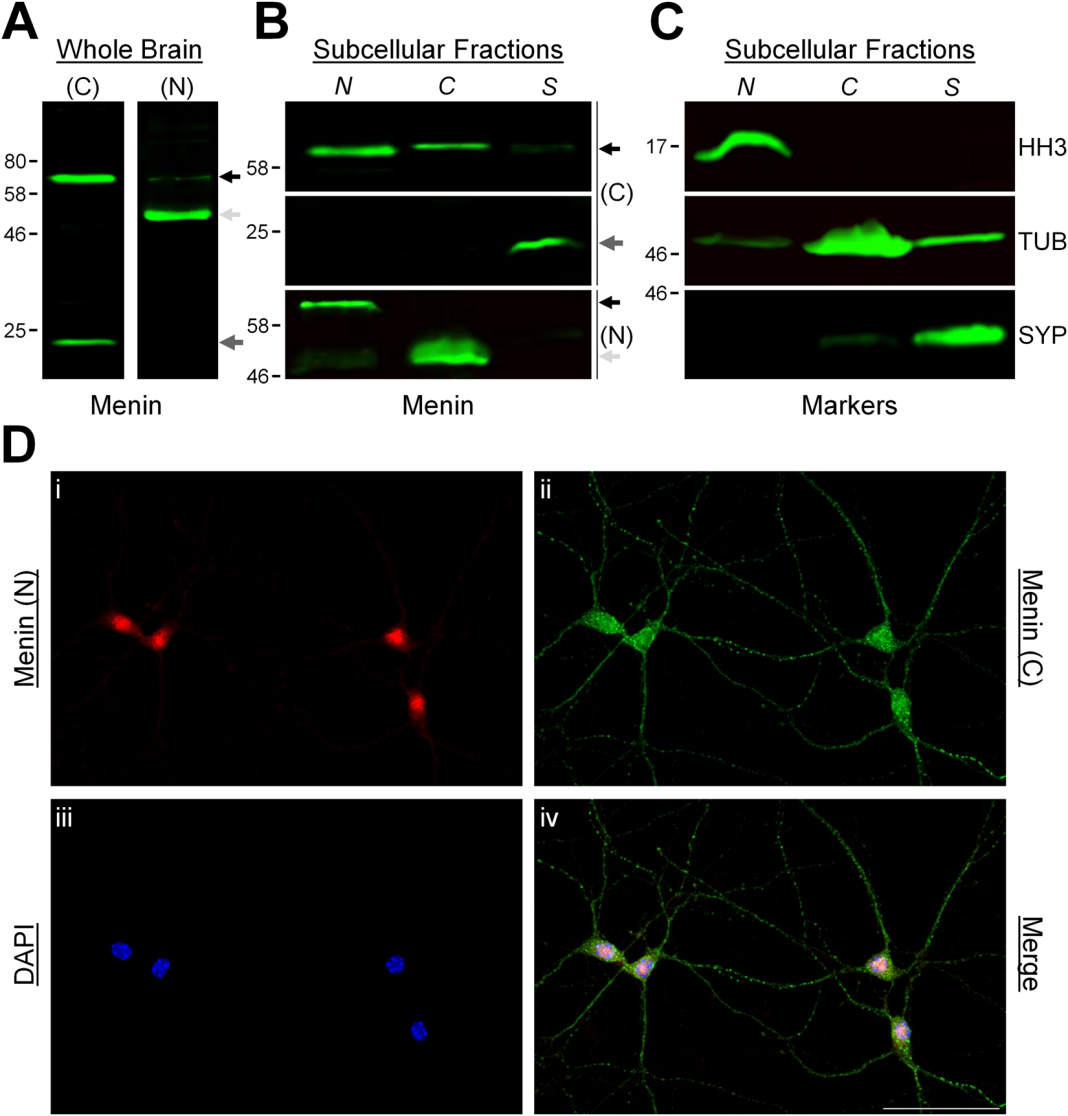
Menin fragments are differentially localized in neurons. (**A**) WB of mouse whole brain protein samples with menin C-terminal ((C); left panel) and N-terminal ((N); right panel) epitope antibodies (n = 3 each, representative blots), depicting full length menin (black arrow), as well as N-terminal (light grey arrow) and C-terminal (dark grey arrow) menin proteolytic fragments. (**B**) WB of subcellular fractions from mouse brain protein samples with menin C-terminal ((C); top and middle panels) and N-terminal ((N); bottom panel) epitope antibodies (n = 6, representative blots). *N* denotes nuclear fraction, *C* denotes cytoplasmic fraction, *S* denotes synaptic fraction. Menin localizes to the nucleus, the C-menin fragment localizes to synaptic membranes, and the N-menin fragment localizes to the cytoplasm. (**C**) As in (**B**), the markers histone H3 (HH3; nuclear marker, top panel), β-tubulin (TUB; cytoplasmic marker, middle panel), and synaptophysin (SYP; synaptic marker, bottom panel), are shown to verify the subcellular fractions. (**D**) ICC localization of menin in hippocampal cultures at DIV 7 (n = 13 images, 4 independent samples, representative image), with N-terminal (i) and C-terminal (ii) epitope antibodies, and the nuclear stain DAPI (iii), (iv) shows merged channels. α-N-terminal menin signals are restricted to the nuclear and perinuclear region, α-C-terminal menin signals also exhibit punctate localization along neurites. Scale bar, 50 μm. See also Figs S1 and S2.

Menin contains both nuclear localization signals and nuclear export signals, which are known to shuttle menin in and out of the nucleus in response to various signal transduction cascades^31, 32^. We have recently characterized the proteolytic cleavage of invertebrate *L*-menin by calpain at an evolutionarily conserved consensus site^6^. The calpain consensus sequence is found in menin orthologues from *Drosophila* to human, suggesting that there has been considerable evolutionary constraint on menin proteolytic cleavage (see Fig. S1), and that these fragments may perform necessary biological functions that are yet to be fully characterized. In *Lymnaea* central neurons, we found that *L*-menin and its amino (N)-terminal and carboxyl (C)-terminal fragments exhibited differential subcellular localization, and coordinated the transcriptional expression and functional clustering of *L*-nAChRs during cholinergic synaptogenesis^6^. As a synaptogenic function for menin is found from invertebrates to vertebrates^4–8^, we hypothesized that a similar mechanism of action underlies its effects in mammalian neurons. To this end, we performed Western blot (WB) analysis and subcellular fractionation on protein samples from mouse brain tissue. Cleavage at the conserved calpain consensus site would produce mouse menin fragments with predicted molecular weights of 48.5 kDa (N-terminal menin fragment; N-menin) and 19 kDa (C-terminal menin fragment; C-menin). Using commercial menin antibodies against C-terminal and N-terminal epitopes (see Fig. S2), we detected bands of appropriate molecular weight for menin (67.5 kDa), as well as faster migrating lower bands of ~19 kDa (α-C-terminal menin) and ~48.5 kDa (α-N-terminal menin) (Fig. 1A; n = 3 each), which correspond to both the predicted banding patterns of calpain cleavage fragments and the molecular weight of full length menin. We then performed subcellular fractionation and WB analysis to determine whether differential subcellular distribution of menin and its proteolytic fragments occurs in the mouse CNS. Microsomes exhibited full length menin predominantly in the nuclear fraction, the N-menin fragment in the cytoplasmic fraction, and the C-menin fragment in the synaptic fraction (Fig. 1B,C; n = 6).

We next performed immunocytochemistry (ICC) on primary hippocampal cultures (DIV 7) to determine whether the patterns of menin subcellular distribution identified by WB are also observed in neurons. ICC with the N- and C-terminal menin antibodies showed both N- and C-terminal α-menin signals in neuronal nuclei, indicative of full length menin. α-N-terminal signals were restricted to the nuclear and perinuclear compartment, whereas α-C-terminal signals also exhibited punctate staining along neurites (Fig. 1D; n = 13). This segregation of menin epitope immunoreactivity in neurons is consistent with the results of WB fractionation, and supports the proteolytic cleavage of menin^6^. Taken together, these observations suggest that menin and its fragments mediate distinct molecular functions via differential subcellular localization.

**Figure 2:**
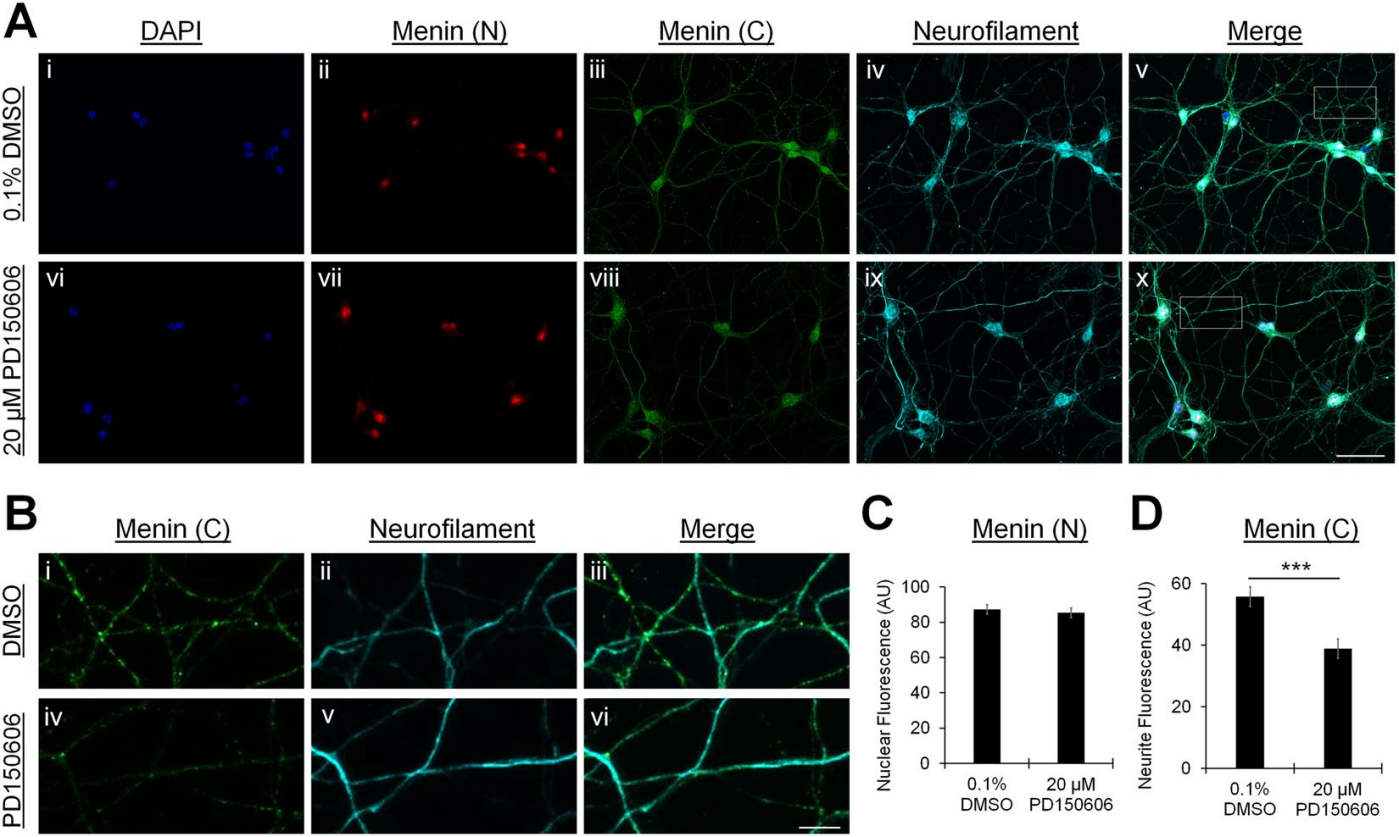
Menin is cleaved by calpain. (**A**) ICC localization of menin in hippocampal cultures at DIV 7, cultured in the presence of 0.1% DMSO vehicle control (i–v) or 20 μM PD150606 (vi–x), a cell permeable calpain inhibitor (n = 9 images, 3 independent samples each, representative images). Cells were labeled with the nuclear stain DAPI (i,vi), menin N-terminal (ii,vii) and C-terminal (iii,viii) epitope antibodies, and a neurofilament antibody (iv,ix), (v,x) shows merged channels. Scale bar, 50 μm. (**B**) Enlarged region of interest (ROI) depicting neurite structure from the boxed region shown in Av (i–iii) and Ax (iv–vi). Scale bar, 10 μm. (**C**,**D**) Summary data, fluorescence intensity of the nuclear α-N-menin signal was unaffected (**C**; ROIs: 0.1% DMSO n = 67, 20 μM PD150606 n = 59), whereas the neurite α-C-menin signal was reduced upon calpain inhibition (**D**; ROIs: n = 27 each). ***Statistical significance (independent t-test), *P* < 0.001. See also Table S1 and Figs S1 and S2.

To verify that the α-C-terminal menin signal in neurites depicts a calpain-dependent proteolytic fragment, neurons were cultured in the presence of a cell-permeable calpain inhibitor (20 μM PD50606) or vehicle control (0.1% DMSO), and processed for ICC as above (DIV 7). We analyzed the fluorescence intensity of nuclear α-N-terminal menin signals (defined by the nuclear stain DAPI) and neurite α-C-terminal menin signals (defined by a neurofilament immunolabel). Consistent with the predicted calpain cleavage site, the appearance of C-menin puncta in neurites was attenuated by calpain inhibition (Fig. 2A,B; n = 9 each). Relative to 0.1% DMSO vehicle control, nuclear α-N-terminal menin fluorescence was unaffected by 20 μM PD50606 (Fig. 2C; *P* = 0.646, Mann-Whitney U test; see Table S1), whereas neurite α-C-terminal menin fluorescence was significantly reduced (Fig. 2D; *P* < 0.001, unpaired t-test). These data demonstrate that menin is proteolytically cleaved by calpain and that the C-terminal proteolytic fragment is targeted to neurites. It should also be noted that we found menin expression in hippocampal cultures to be confined to neurons, as both α-N- and α-C-terminal signals were absent in the glial layer that is prominent in mature cultures (see Fig. S3), suggesting that menin may serve neuron-specific functions in the CNS.

### The menin C-terminal fragment colocalizes with α7 subunit-containing nAChRs at presynaptic terminals

**Figure 3:**
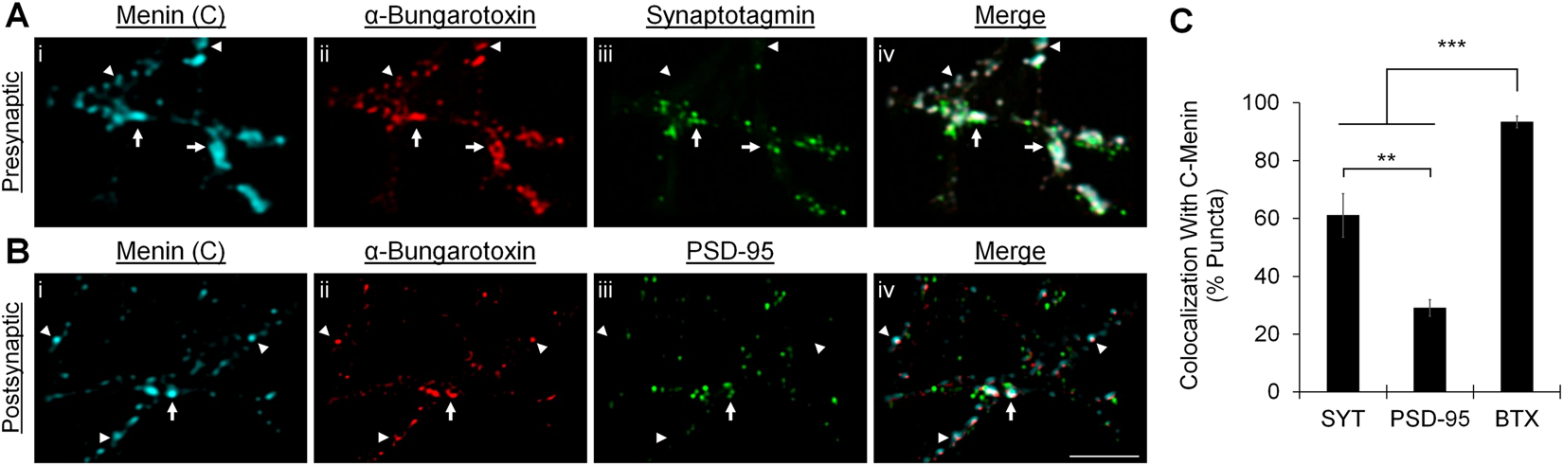
The C-terminal menin fragment colocalizes with α7 subunit-containing nAChRs at presynaptic terminals. (**A**) Super resolution image of a synaptic ROI at DIV 7 (n = 9 images, 2 independent samples, representative image), cells were labeled with α-C-terminal menin (i), α-bungarotoxin (ii; α7-nAChR), and α-synaptotagmin (iii; presynaptic marker), (iv) shows merged channels. (**B**) As in (**A**), only labeled with α-PSD-95 (iii; postsynaptic marker) (n = 10 images, 2 independent samples, representative image). Arrowheads, extrasynaptic colocalization of C-menin and α7-nAChRs; arrows, synaptic colocalization with synaptotagmin (**A**) or PSD-95 (**B**). Scale bar, 2 μm. (**C**) Summary data, incidence of colocalization of synaptotagmin (SYT; presynaptic), PSD-95 (postsynaptic), and α-bungarotoxin (BTX; α7-nAChR) puncta with C-menin. Asterisks, statistical significance (Kruskal-Wallis test); **P < 0.01. ***P < 0.001. See also Table S2 and Fig. S4.

While cholinergic innervation is absent in hippocampal cultures, α-bungarotoxin (α-BTX) sensitive nAChRs, which contain the α7 subunit^33^, localize to glutamatergic presynaptic terminals, and their activation induces presynaptic facilitation^34, 35^. This suggests that the necessary components of cholinergic postsynaptic machinery underlying the functional clustering of nAChRs are expressed in hippocampal neurons notwithstanding the absence of presynaptic input. To determine whether the punctate localization of the C-menin fragment along neurites might indicate the synaptic clustering of nAChRs in mouse hippocampal neurons, we used super resolution fluorescence microscopy on DIV 7 cultures labeled with fluorophore-tagged α-BTX, and antibodies against the C-terminal menin epitope, as well as the synaptic vesicle protein synaptotagmin (SYT) as a presynaptic marker, or the glutamatergic scaffold postsynaptic density 95 (PSD-95) as a postsynaptic marker (Fig. 3A,B; n ≥ 9). We identified a nearly 1:1 incidence of colocalization between C-menin and α-BTX labeled puncta. The degree of colocalization of C-menin with nAChRs was significantly higher than the colocalization with either the presynaptic marker SYT or the postsynaptic marker PSD-95 (Fig. 3C; *P* < 0.001, Kruskal-Wallis test; see Table S2 and Fig. S4). C-menin was also found to be more closely associated with presynaptic rather than postsynaptic sites between hippocampal neurons (*P* = 0.001). Considering our previous observations from invertebrate neurons that the synaptogenic effects of *L*-menin occur at the postsynapse^4–6^, the low correlation of C-menin and the glutamatergic postsynaptic density scaffold PSD-95 suggests that menin does not function as a ubiquitous synaptogenic factor, but that its molecular actions may be specific to the postsynaptic machinery of nAChRs. Furthermore, the high degree of correlation of C-menin and α-BTX with SYT at presynaptic terminals suggests that the molecular actions of C-menin in mammalian central neurons may be specific to the axonal targeting of α7 subunit-containing nAChRs.

### Menin mediates subunit-selective transcriptional regulation of nAChR α5

**Figure 4:**
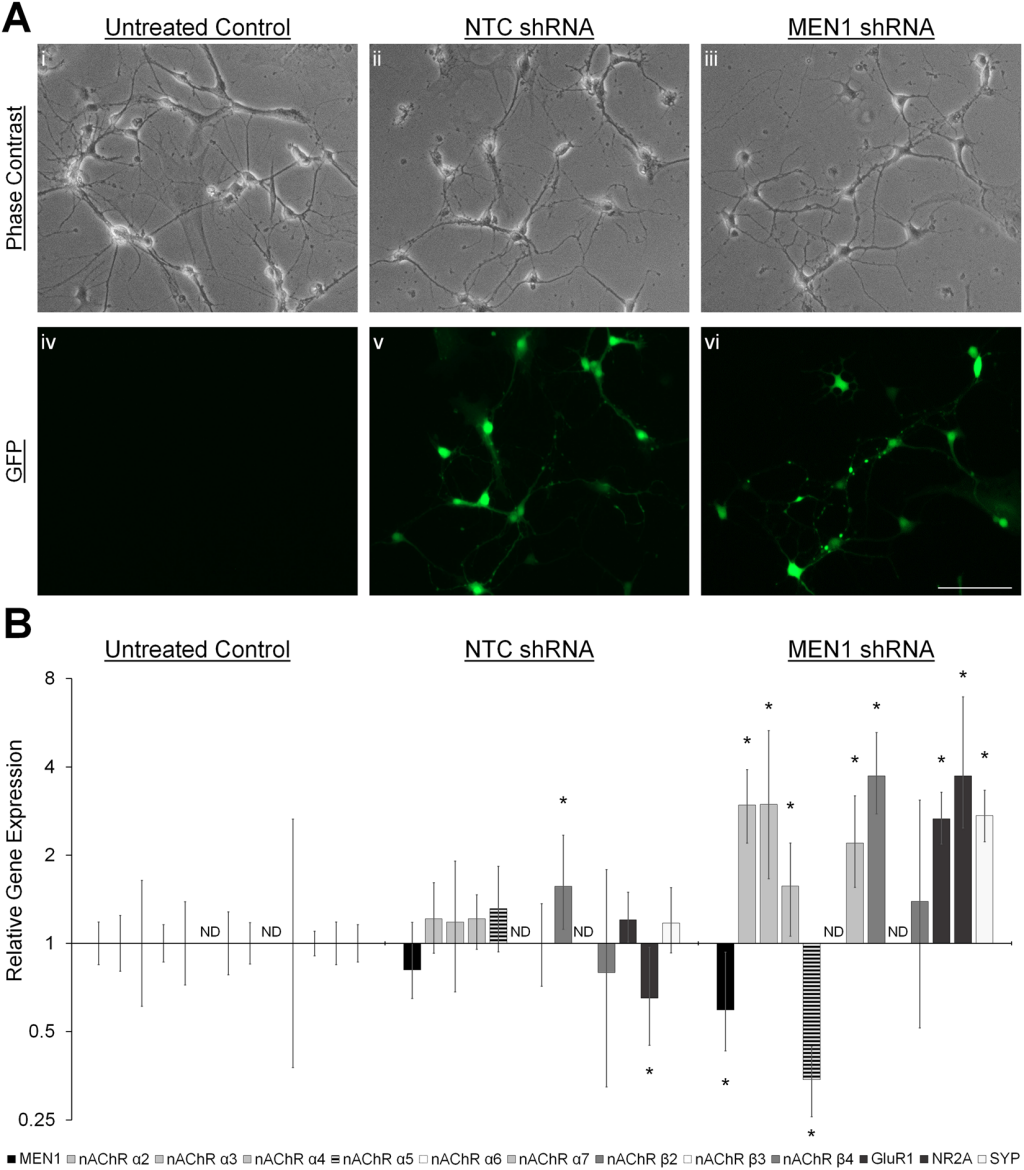
Menin mediates subunit-specific transcriptional regulation of nAChR α5. (**A**) Live cell phase contrast (**i-iii**) and GFP fluorescence (**iv-vi**) images of untreated control (i,iv), NTC shRNA (ii,v), and *MEN1* shRNA (iii,vi) lentivirus transduced hippocampal cultures at DIV 7 (n = 18 images, 6 independent samples each, representative images). Scale bar, 100 μm. (**B**) Summary data, fold change gene expression in hippocampal cultures at DIV 7, relative to untreated control, determined by qPCR (n = 6, 2 independent experiments each, triplicate replicates). *MEN1* knockdown reduces nAChR α5 expression. ND, qPCR signal not detected. *Statistical significance (pair wise fixed reallocation randomization test), *P* < 0.05–0.001. See also Table S3.

Menin is largely regarded as a nuclear protein^36^, and its dual role as a transcriptional activator and transcriptional repressor, acting through distinct complexes, is well documented in the cancer literature^37^. We recently reported that *L*-menin induces subunit-selective transcriptional upregulation of *L*-nAChRs^6^ in invertebrate central neurons, and we hypothesized that menin may exhibit similar transcriptional effects in mammalian central neurons. To this end, hippocampal cultures were transduced with lentivirus constructs encoding non-target control (NTC) or *MEN1* shRNA at DIV 1 (Fig. 4A), and RNA samples were collected from untreated control, NTC shRNA- and *MEN1* shRNA-transduced samples at DIV 7 for qPCR analysis (n = 6, triplicate replicates; see Table S3). We assayed for neuronal nAChR subunits α2-7 and β2-4, the glutamate receptor subunits GluR1 (AMPA-type) and NR2A (NMDA-type), as well as the synaptic vesicle protein synaptophysin (SYP). NTC shRNA had a minimal effect on transcriptional regulation, inducing a slight increase in nAChR β2 (*P* = 0.014, pair wise fixed reallocation randomization test) and a slight decrease in NR2A (*P* = 0.019) transcript abundance. *MEN1* knockdown (Fig. 4B; *P* = 0.009), however, induced a subunit-specific reduction in nAChR α5 transcript levels (*P* = 0.002), whereas most other transcripts were elevated (*P* < 0.05–0.001). These data suggest, on the one hand, that nAChR α5 subunit expression requires menin-dependent transcriptional activation, and on the other hand, that the remaining transcripts are regulated by menin-dependent transcriptional repression^11^.

**Figure 5:**
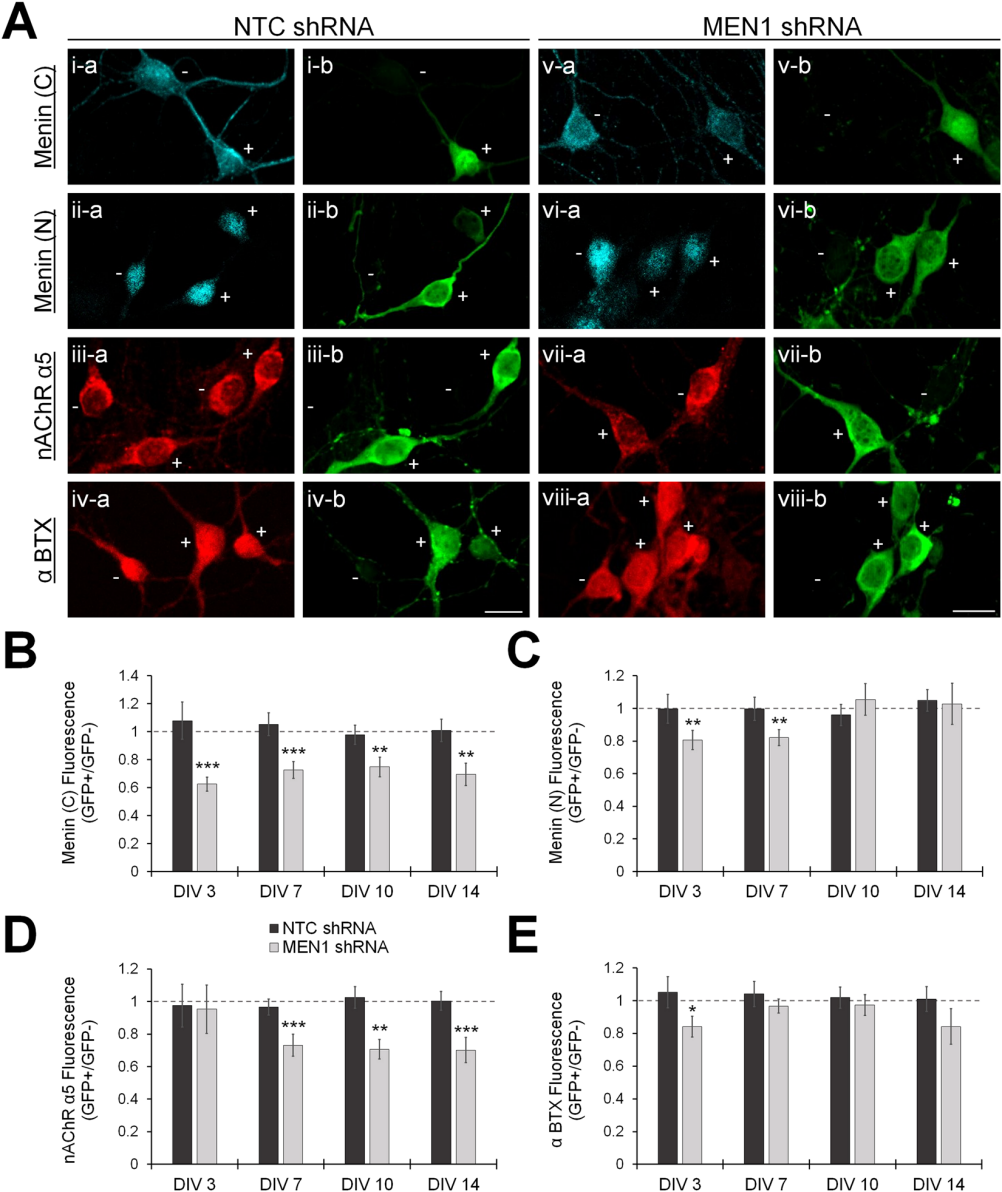
Menin knockdown reduces nAChR α5 subunit expression. (**A**) ICC characterization of menin and nAChR protein expression in neuronal soma from NTC shRNA and *MEN1* shRNA lentivirus transduced hippocampal cultures (n ≥ 4 images, ≥2 independent samples; see Table S4; representative images, DIV 7). Untransduced neurons were GFP negative (−) and transduced neurons were GFP positive (+). The expression of menin was determined with C-terminal (i,v) and N-terminal (ii,vi) epitope antibodies, and nAChRs with a nAChR α5 antibody (iii,vii), or fluorophore-tagged α-BTX (iv,viii; α7-nAChR). ICC labels are shown in **a** (left panels), and GFP fluorescence is shown in b (right panels). Scale bars, 20 μm. (**B–E**) Summary data, normalized somal fluorescence intensity of α-C-terminal menin (**B**), α-N-terminal menin (**C**), α-nAChR α5 (**D**), and α-BTX (**E**), in GFP + neurons relative to GFP- neurons at DIV 3–14 (ROIs: n ≥ 12 each; see Table S4). Dashed lines represent a 1:1 ratio indicating no change. Asterisks, statistical significance (independent t-test or Mann-Whitney U test); *P < 0.05. **P < 0.01. ***P < 0.001.

We next used ICC fluorescence intensity analysis to verify menin knockdown and nAChR α5 downregulation in *MEN1* shRNA-transduced cultures at the protein level. Images were obtained from NTC shRNA- and *MEN1* shRNA-transduced cultures from regions containing both lentivirus-transduced (GFP+) and untransduced (GFP-) neurons. Somal fluorescence values were measured from GFP+ and GFP- neurons labeled with C-terminal menin, N-terminal menin or nAChR α5 antibodies, or fluorophore-tagged α-BTX (Fig. 5A; n ≥ 12 each; DIV 3–14, representative images at DIV 7). NTC shRNA-transduced neurons exhibited a GFP+/GFP− normalized fluorescence ratio of approximately 1:1 for α-C-terminal menin, α-N-terminal menin, α-nAChR α5 and α-BTX at all time points (Fig. 5B–E; *P* > 0.05, unpaired t-test or Mann-Whitney U test; see Table S4). In *MEN1* shRNA-transduced neurons, α-C-terminal menin fluorescence was significantly reduced in GFP + relative to GFP- neurons at DIV 3–14 (Fig. 5B; *P* < 0.01–0001). Intriguingly, α-N-terminal menin fluorescence was reduced at DIV 3–7 (Fig. 5C; *P* < 0.01), but recovered at DIV 10–14 (*P* > 0.05), suggesting that the protein stability of menin, N- and C-menin fragments may be regulated independently in response to changes in menin intracellular levels. nAChR α5 fluorescence was reduced at DIV 7–14 (Fig. 5D; *P* ≤ 0.001) but not at DIV 3 (*P* > 0.05), suggesting that nAChR α5 downregulation follows *MEN1* knockdown with a delay, which likely reflects the half-life of existing α5 subunit transcripts and protein. α-BTX fluorescence was reduced at DIV 3 (Fig. 5E; *P* < 0.05), but was unchanged at DIV 7–14 (*P* > 0.05), despite upregulation of the α7 transcript observed at DIV 7 (see Fig. 4). These observations suggest that nAChR α5 subunit expression requires menin-dependent transcriptional activation, and also that the transcripts governed by menin-dependent transcriptional repression, such as nAChR α7, may not exhibit comparable changes in protein abundance in response to menin perturbations.

### The menin C-terminal fragment regulates the presynaptic clustering of α7 subunit-containing nAChRs

**Figure 6:**
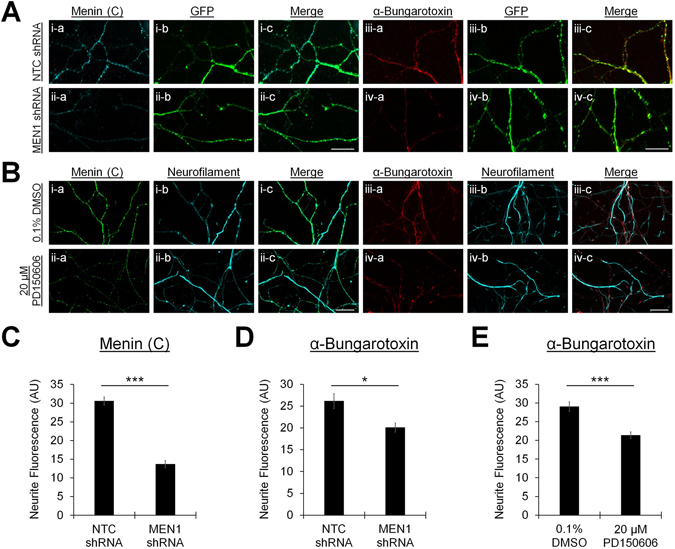
C-menin mediates neurite localization of α7 subunit-containing nAChRs. (**A**,**B**) ICC characterization of the neurite localization of C-menin and α7-nAChRs in NTC shRNA and *MEN1* shRNA lentivirus transduced hippocampal cultures (**A**; n = 18 images, 5 independent samples each; representative images, DIV 7) or hippocampal cultures maintained in the presence of 0.1% DMSO or 20 μM PD150606 (**B**; n = 12 images, 2 independent samples each; representative images, DIV 7). Cells were labeled with the menin C-terminal epitope antibody (i-ii-a) or fluorophore-tagged α-BTX (iii-iv-a), and neurites were visualized with GFP (**A**i-iv-b) or neurofilament (**B**i-iv-b) antibodies, (i-iv-c) shows merged channels. Scale bars, 20 μm. (**C**,**D**) Summary data, the α-C-menin signal in neurites was reduced upon *MEN1* knockdown (**C**), which coincides with a reduction of the neurite α-BTX signal (**D**). (**E**) Summary data, reduction of C-menin abundance by calpain inhibition (see Fig. 2D) coincides with a reduction of the neurite α-BTX signal (ROIs: n ≥ 36 each; see Table S5), indicating that the synaptic targeting of α7-nAChRs requires the menin C-terminal fragment. Asterisks, statistical significance (Mann-Whitney U test); *P < 0.05. ***P < 0.001.

Considering that the menin C-terminal fragment colocalized extensively with α7-nAChRs in neurites (see Fig. 3), we next sought to determine whether the C-menin fragment was indeed required for the targeting of α7-nAChRs to synaptic sites. The fluorescence intensity of α-BTX was first measured in the neurites of neurons transduced with NTC shRNA or *MEN1* shRNA encoding lentivirus (Fig. 6A; n = 18 each), or neurons cultured in the presence of 0.1% DMSO or 20 μM PD150606 (Fig. 6B; n = 12 each). Cells were processed for ICC (DIV 7) and labeled with α-C-menin, α-BTX, and α-GFP or α-neurofilament, which were used to determine the location of neurites. Relative to NTC shRNA, *MEN1* shRNA reduced the fluorescence intensity of α-C-terminal menin as well as α-BTX signals along neurites (Fig. 6C,D; *P* < 0.001 and *P* = 0.038, respectively, Mann-Whitney U test; see Table S5). Calpain inhibition by 20 μM PD150606, which reduced the abundance of the C-terminal menin fragment (see Fig. 2), also reduced the fluorescence intensity of α-BTX signals along neurites relative to 0.1% DMSO vehicle control (Fig. 6E; *P* < 0.001, Mann-Whitney U test), indicating that α7-nAChR targeting by menin is dependent upon the C-terminal proteolytic fragment.

**Figure 7:**
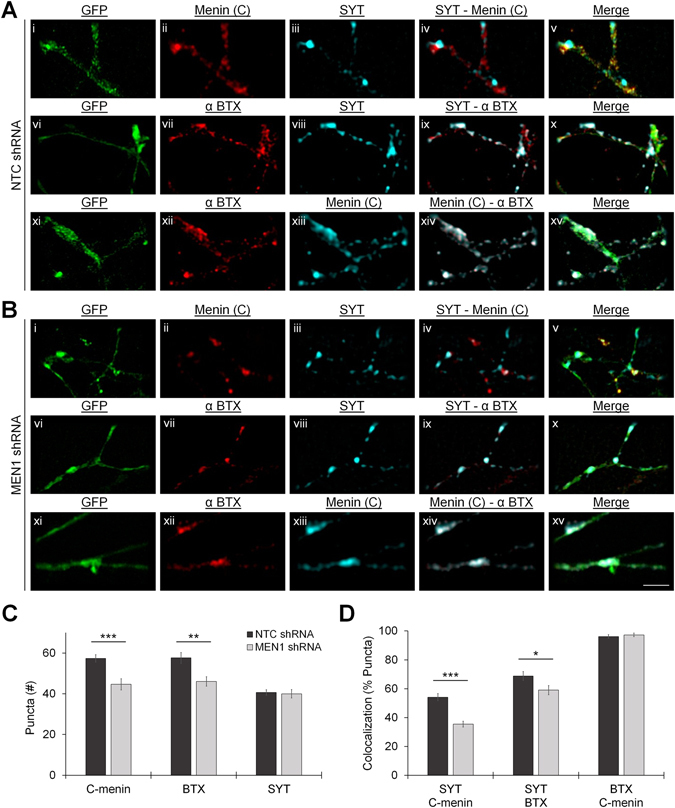
Menin knockdown reduces the presynaptic clustering of α7 subunit-containing nAChRs. (**A**,**B**) Super resolution images of C-menin, α-BTX, and SYT puncta in GFP+ synaptic ROIs from NTC shRNA (**A**) and *MEN1* shRNA (**B**) lentivirus transduced hippocampal cultures at DIV 7 (n ≥ 10 images, 2 independent samples each, representative images). Cells were labeled with C-terminal menin and SYT antibodies (i–v; NTC: n = 16, *MEN1*: n = 14), fluorophore-tagged α-BTX and a SYT antibody (vi–x; NTC: n = 16, *MEN1*: n = 15), or α-BTX and a C-terminal menin antibody (xi–xv; NTC: n = 11, *MEN1*: n = 10). (i,vi,xi) shows GFP fluorescence, (ii,xiii) shows α-C-terminal menin fluorescence, (vii,xii) shows α-BTX fluorescence, (iii,viii) shows α-SYT fluorescence, (iv,ix,xiv) shows SYT/C-menin/α-BTX merged channels, (v,x,xv) shows channels merged with GFP. Scale bar, 2 μm. (**C**) Summary data, mean number of α-C-terminal menin, α-BTX, and α-SYT puncta in super resolution images. (**D**) Summary data, incidence of colocalization of SYT/C-menin/α-BTX puncta in super resolution images. Asterisks, statistical significance (independent t-test or Mann-Whitney U test); *P < 0.05. **P < 0.01. ***P < 0.001. See also Table S6.

We then performed super resolution fluorescence microscopy on NTC shRNA- and *MEN1* shRNA-transduced neurons to determine whether the reduction of α-C-menin and α-BTX fluorescence intensity along neurites encompasses a deficit of C-menin and α7-nAChR clustering to presynaptic sites (Fig. 7A,B; DIV 7; n ≥ 10; see Table S6). DIV 7 cultures were labeled with (i) α-C-menin and α-SYT, (ii) α-BTX and α-SYT, or (iii) α-BTX and α-C-menin, and images were acquired from GFP+ regions of interest (ROIs). Relative to NTC control, *MEN1* knockdown significantly reduced the number of both C-menin (Fig. 7C; *P* < 0.001, unpaired t-test) and α-BTX puncta (*P* = 0.002, Mann-Whitney U test), but had no effect on the number of SYT puncta in neurites (*P* = 0.790, unpaired t-test). Furthermore, we found that the frequency of colocalization of SYT with C-menin (Fig. 7D; *P* < 0.001, Mann-Whitney U test) and α-BTX (*P* = 0.033, unpaired t-test) was significantly reduced by *MEN1* knockdown, but the incidence of C-menin and α-BTX colocalization was unaffected (*P* = 0.430, Mann-Whitney U test).

**Figure 8:**
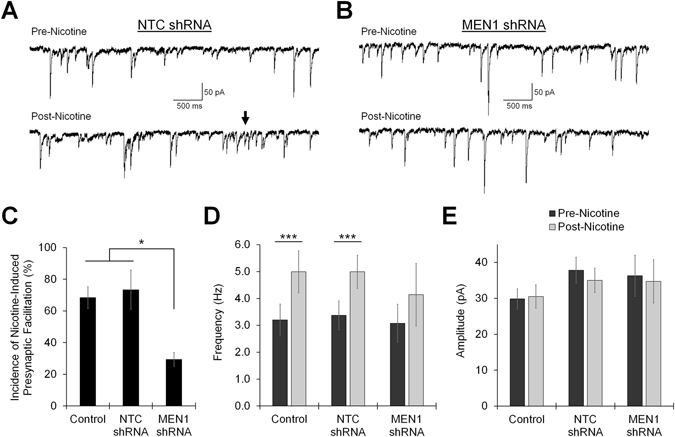
Menin knockdown reduces nicotine-induced presynaptic facilitation. (**A**,**B**) Representative patch clamp traces recorded from GFP+ hippocampal pyramidal neurons at DIV 10–14 (n ≥ 15, 3 independent experiments, representative traces). The mean frequency and amplitude of mEPSCs was analyzed before (Pre-nicotine, 10 s; top trace) and after (Post-nicotine, 10 s; bottom trace) the nicotine pulse (10 μM, 250 ms, 10 PSI; not shown). nAChR-mediated increase in mEPSC frequency (e.g. arrow) is observed in a GFP+ neuron from NTC-shRNA expressing cultures (**A**), but is absent in a GFP+ neuron from *MEN1*-shRNA expressing cultures (**B**). (**C**) Summary data, incidence of nicotine-induced presynaptic facilitation in neurons from untreated control (n = 13/19), NTC shRNA (n = 11/15; GFP+) and *MEN1* shRNA (n = 5/17; GFP+) transduced hippocampal cultures (post/pre-nicotine relative mEPSC frequency ≥1 ± SEM; see Fig. S5 and Table S8). *Statistical significance (Chi-squared test), *P* < 0.05. (**D**) Summary data, mean frequency of mEPSCs before (pre) and after (post) the nicotine pulse in the population of neurons from untreated control (n = 13), NTC shRNA (n = 11; GFP+) and *MEN1* shRNA (n = 5; GFP+) transduced cultures that exhibit nicotine-induced presynaptic facilitation. ***Statistical significance (paired t-test)*, P* < 0.001. (**E**) Summary data, mean amplitude of mEPSCs, as in (**D**). See also Tables S7–S9 and Figs S5–S7.

The application of nicotine to hippocampal neurons both in slice preparations and in culture is known to increase the frequency of spontaneous miniature excitatory postsynaptic currents (mEPSCs) via calcium influx through activated nAChRs, which facilitates neurotransmitter release by increasing synaptic vesicle release probability^34^. Patch-clamp recordings were next made from pyramidal neurons in untreated control, NTC shRNA- and *MEN1* shRNA-transduced cultures (DIV 10–14; transduced at DIV 1) to evaluate whether the mislocalization of presynaptic α7-nAChRs induced by menin knockdown disrupts nicotine-induced presynaptic facilitation (Fig. 8A,B; n ≥ 15; see Tables S7–S9 and Figs S5–S7). We recorded from GFP+ neurons to determine whether other aspects of postsynaptic function (such as glutamate receptor clustering) may also be affected by menin perturbations. Relative to untreated control, we found that NTC shRNA and *MEN1* shRNA expression did not alter the baseline amplitude or frequency of mEPSCs, suggesting that glutamatergic synaptogenesis proceeded normally upon menin knockdown (*P* = 0.844 and *P* = 0.241, one-way ANOVA; see Table S7 and Fig. S5). In untreated control cultures, 13 of 19 neurons (68.42%) exhibited an increase in mEPSC frequency in response to the application of 10 μM nicotine (Fig. 8C; post-nicotine/pre-nicotine frequency ≥1 ± SEM; see Table S8 and Fig. S5). Similarly, 11 of 15 neurons (73.33%) in NTC shRNA-transduced cultures exhibited nicotine-induced presynaptic facilitation (*P* = 0.755, Chi-squared test), whereas only 5 of 17 neurons (29.41%) in *MEN1* shRNA-transduced cultures exhibited a similar response (*P* = 0.019). Of the neurons exhibiting an increase in mEPSC frequency in response to nicotine, we found that the mean mEPSC frequency was significantly increased in untreated control (Fig. 8D; *P* < 0.001, paired t-test; see Table S9) and NTC shRNA-transduced cultures (*P* < 0.001), but not *MEN1* shRNA-transduced cultures (*P* = 0.130). The mean amplitude of mEPSCs was not affected by the application of nicotine (Fig. 8E; *P* ≥ 0.130, paired t-test; see Tables S7–S9 and Fig. S5), or amongst treatment groups (*P* ≥ 0.543, one-way ANOVA). Taken together, these data demonstrate that the menin C-terminal fragment influences the clustering of α7-nAChRs at presynaptic terminals, and that menin’s synaptogenic function is specific to the regulation of nAChR channels.

## Discussion

Whereas the function of menin in cancer biology and transcriptional regulation has been well studied^11^, its role in the nervous system is yet to be fully realized. In the present study, we sought to identify the molecular mechanisms underlying the synaptogenic function of menin in the mammalian CNS. Here, we provide the first evidence that transcriptional regulation of the nAChR α5 subunit, and the functional clustering of α7 subunit-containing nAChRs involved in presynaptic facilitation, are dependent upon the molecular actions of menin. Our data furthermore support a conserved function for the calpain-dependent menin C-terminal proteolytic fragment in the clustering of neuronal nAChRs, and indicate that this effect is limited to a specific class of neurotransmitter receptors.

### Menin proteolytic cleavage and differential subcellular distribution

Studies using the α-C-terminal menin epitope antibody routinely detect a lower band of ~19 kDa in Western blots of protein samples from various mammalian preparations^36, 38, 39^, although this has been largely disregarded in the literature as a supposedly non-specific antibody interaction. A recent report from our group, however, characterized the proteolytic cleavage of invertebrate *L*-menin at an evolutionarily conserved calpain consensus site, and provided the first indication of a functional significance for the C-terminal menin fragment, in mediating the synaptic clustering of *L*-nAChRs^6^. In the present study, we show that (i) menin in the mouse CNS is similarly cleaved by calpain, (ii) menin and its proteolytic fragments exhibit distinct patterns of subcellular localization, and (iii) the C-menin fragment clusters α7-nAChRs at glutamatergic presynaptic terminals. These observations suggest that the C-menin-dependent clustering of neuronal nAChRs is a specialized cellular function of menin. However, calpain expression is ubiquitous and the appearance of a C-terminal menin immunoreactive fragment has been described in multiple experimental preparations and tissues, suggesting that the function of C-menin may not be limited to the clustering of nAChRs in neurons. For instance, C-terminal menin immunoreactivity has been found to localize to the membrane of pancreatic islets^40^, and pancreatic β-cells have been reported to express nAChRs^41^. In line with our present findings, this localization could be indicative of the C-menin fragment associated with nAChRs, and suggests that the functional clustering of nAChRs by the C-menin proteolytic fragment may also occur in non-neuronal tissues.

### Menin-dependent transcriptional regulation

Menin is a multifunction transcriptional regulator, acting in complexes with numerous transcriptional activators, transcriptional repressors, or cell signaling proteins^11, 37^. However, the impact of menin-dependent transcriptional regulation on neuronal and synaptic function is currently not well understood. We have recently reported that *L*-menin induces subunit-specific transcriptional upregulation of *L*-nAChRs in invertebrate *Lymnaea* neurons^6^. In the present study, we show that menin knockdown in mouse hippocampal cultures downregulates nAChR α5 transcription, but otherwise induces the transcriptional upregulation of multiple synapse-associated gene products, including other nAChR subunits, glutamate receptor subunits, and synaptophysin. This subunit-specific transcriptional regulation of nAChR α5 may be due to (i) differences in gene promoter sequences, with α5 binding menin transcriptional activator complexes, whereas the promoter sequences of other genes may bind menin transcriptional repressor complexes, or (ii) the transcriptional polarity of the α5 gene, which is opposite to other nAChR subunits^42^ and may differentially affect transcriptional activation. Considering the observation reported here that menin expression in hippocampal cultures is restricted to neurons, a previous report that nAChR α5 expression is found in neurons, but not glia^43^, supports the role of menin in the transcriptional activation of nAChR α5.

The accumulation of N-terminal menin immunoreactivity alongside the sustained reduction of C-terminal menin immunoreactivity upon *MEN1* knockdown is an intriguing observation that supports an autoregulation function for *MEN1*/menin^44^, and may contribute to further insights into the cellular homeostatic function of menin as a tumor suppressor. The accumulation of N-menin, but not C-menin, immunoreactivity in response to *MEN1* knockdown suggests that the stability of menin and the two proteolytic protein fragments is differentially regulated, perhaps through a feedback loop involving cell signaling cascades, protein-protein interactions, transcriptional regulation, or posttranslational modifications^11, 31, 37, 45, 46^. This observation furthermore supports the hypothesis that menin and its proteolytic fragments perform distinct molecular functions. Consistent with this notion, we have previously reported that in *Lymnaea* CNS neurons, the N-terminal, but not the C-terminal *L*-menin fragment induces transcriptional upregulation of the endogenous *L*-*MEN1* gene, indicating a positive feedforward system mediated by the N-menin fragment^6^. Conversely, multiple *cis*-regulatory elements have been identified in the human *MEN1* gene promoter, and *MEN1* promoter activity has been found to decrease in response to menin overexpression, indicating a negative feedback system mediated by full length menin^44^. While we show here that the mouse N-menin fragment localizes to the cytoplasm, this does not necessarily exclude the existence of N-menin-dependent transcriptional regulation of *MEN1* and/or other genes in response to reduced intracellular levels of menin. For example, translocation of menin to the cytoplasm has been found to shuttle another transcriptional regulator, β-Catenin, out of the nucleus to regulate its transcriptional activity^31^. Ultimately, these findings highlight the complex transcriptional networks underlying *MEN1* gene induction and the gene targets that are transcriptionally activated or repressed by menin.

### nAChRs on presynaptic terminals: subunit composition, function, and clustering

Cholinergic synaptic transmission in the brain is thought to play a modulatory role in regulating neuronal network activity^47^, and the facilitation of neurotransmitter release mediated by nAChR activation has been demonstrated for most classical neurotransmitters employed in the CNS^19^. These observations are consistent with the targeting of nAChRs to presynaptic terminals, and the actions of nAChRs as calcium-conducting cation channels in increasing the rate of quantal release through a spatiotemporal calcium-dependent mechanism analogous to paired-pulse facilitation^34^. Although the involvement of α-BTX sensitive (i.e. α7 subunit-containing) nAChRs in nicotine-induced facilitation of glutamatergic transmission is well documented^34, 47^, the subunit composition of native presynaptic nAChRs is currently not well defined. The functional and pharmacological characteristics of presynaptic α7-containing nAChRs are distinct from those of homomeric α7 channels expressed in heterologous expression systems^48^, indicating that the native receptors may be non-homomeric α7-containing channels^49^. Whereas α-BTX blockade normally eliminates nicotine-induced presynaptic facilitation, knockdown of the α7 subunit has been found to result in the emergence of α-BTX-insensitive facilitation, suggesting that either the trafficking of non-α7 nAChRs to presynaptic sites occurs as compensation, or the α7 subunit is one component of the presynaptic nAChR channels normally involved in presynaptic regulation^47^. In the present study, we show that *MEN1* knockdown in hippocampal cultures reduces (i) nAChR α5 subunit expression, (ii) the clustering of α7-nAChRs to presynaptic sites, and (iii) nicotine-induced presynaptic facilitation. Together, our observations suggest that the loss of nAChR-dependent facilitation involves a deficit of α7-nAChR clustering at presynaptic sites, and also raise the possibility that menin may regulate nAChR channel function by influencing subunit composition. The modulatory α5 subunit has previously been reported to enhance the ACh sensitivity, Ca^2+^ permeability, and conductance of nAChR channels^50, 51^. Considering that the existence of heterologous α7-containing nAChRs has been previously suggested^52, 53^, it may be that the α5 subunit is an adjustable component of presynaptic nAChR channels. This notion would furthermore be in agreement with our previous findings on the role of *L-*menin in coordinating the transcriptional upregulation and synaptic clustering of *L*-nAChRs in *Lymnaea* central neurons during cholinergic synaptogenesis^6^.

At the NMJ, high-density clustering of nAChRs is mediated by the molecular scaffold rapsyn^21^. In contrast to post-junctional muscle membranes, which receive only cholinergic innervation, most neurons receive a variety of synaptic inputs and must meet the additional requirement of sorting multiple types of neurotransmitter receptors to appropriate synaptic sites. This feat is usually accomplished by specific molecular interactions and a physical association between the intracellular domain of a given receptor and a dedicated scaffold or sets of scaffold and adaptor molecules. Glutamate receptor clustering, for example, is mediated by interactions between cytoplasmic C-terminal residues either directly with the PDZ domains of PSD-95-type molecular scaffolds (NMDA-type), or indirectly via PDZ domain-containing molecular adaptor proteins GRIP (glutamate receptor-interacting protein) or PICK1 (protein interacting with C kinase) that bind PSD-95 (AMPA-type)^54–56^. Rapsyn, however, apparently does not mediate the synaptic clustering of neuronal nAChRs^28, 57^. While PDZ domain-containing PSD-95 family members have been reported to influence the synaptic clustering of various types of neuronal nAChRs, these molecular scaffolds are not required for neuronal nAChR clustering^58, 59^. Our observations that *MEN1* knockdown reduced the clustering of nAChRs at presynaptic terminals and nicotine-induced presynaptic facilitation would be consistent with a role for the C-menin fragment as a molecular scaffold or adaptor for the synaptic clustering of α7 subunit-containing nAChRs. As pre- and postsynaptic membrane specializations contain multiple elements with PDZ domains, we suspect that the influence of PSD-95-type PDZ scaffolds on neuronal nAChR clustering likely represents a mechanism for nAChR targeting to appropriate sites adjacent to non-cholinergic presynaptic active zones and postsynaptic densities, given the primarily modulatory role of cholinergic synaptic transmission in the CNS.

### The role of menin and nAChRs in synapse formation, plasticity and maintenance

In the spinal cord dorsal horn, synaptic plasticity is a critical step in the emergence of hypersensitivity to normally innocuous stimuli following peripheral nerve injury. The upregulation of menin^7–9^ and nAChR α5 subunit^60^ expression in the spinal cord dorsal horn have both been reported to be required for the development of neuropathic pain after peripheral nerve injury. The transcriptional upregulation of the modulatory α5 subunit by menin, the contribution of α5 to high-conductance nAChR channels^50, 51^, and the enhanced presynaptic clustering of α7-containing nAChRs via C-menin, would all be consistent with the potentiation of nAChR-induced presynaptic facilitation and synaptic hyperexcitability during neuropathic pain. As we found that nAChR α5 gene induction and α7-nAChR clustering are dependent on *MEN1* expression, these observations suggest that the molecular actions of menin tune nAChR function by regulating subunit composition and synaptic clustering, and may thus represent a novel cholinergic mechanism underlying injury-induced synaptic plasticity and the development of neuropathic pain.

In the hippocampus, activation of nAChRs is known to promote synapse formation and plasticity, as well as the survival, maturation and synaptic integration of adult-born neurons^61, 62^. AD is associated with hippocampal pathology, nAChR perturbations, and cholinergic neuron death^17, 63–65^. Recently, reduced expression of the BRCA1 and PTEN tumor suppressors have been implicated in the synaptic deficits observed in AD^66, 67^, contributing to an emerging hypothesis that tumor suppressor dysfunction underlies AD pathophysiology. These mechanisms, however, have not yet been directly linked to the regulation of nAChRs. Given the cholinergic and synaptic plasticity dysfunctions observed in AD, we suspect that the *MEN1*-dependent regulation of nAChR expression and clustering described here may play a role in AD pathophysiology as well.

## Methods

### Animals and Neuronal Cell Culture

Animal procedures were approved by the University of Calgary institutional animal use and care committee, in accordance with the standards established by the Canadian Council on Animal Care. All experiments involving animals were performed in accordance with these regulations. Brains were dissected from 2.5 month old male C57/BL6 mice (Charles River) that were anesthetized with isoflurane and sacrificed by decapitation. Tissue was frozen on dry ice and stored at −80 °C. Dissociated primary hippocampal neuron cultures were prepared from embryonic C57/BL6 mice (Charles River). Pregnant dams were anesthetized with isoflurane and sacrificed by decapitation. Embryonic day 18 embryos were immediately dissected and sacrificed by decapitation. Hippocampi were dissected in 1 × HBSS containing 10 mM HEPES (310 mOsm, pH 7.2), and treated with an enzyme mixture containing papain (50 U/mL), 150 mM CaCl_2_, 100 µM L-cysteine, and 500 µM EDTA in neurobasal medium (NBM) for 20 m at 37 °C, then washed 3x with NBM supplemented with 4% FBS, 2% B27, 1% penicillin-streptomycin and 1% L-Glutamine (GIBCO). Neurons were dissociated by trituration with polished glass Pasteur pipettes, and plated at a low density onto glass coverslips (washed with nitric acid and coated with poly-D-lysine [30 µg/mL; Sigma Aldrich] and laminin [2 µg/mL; Sigma Aldrich]) in costar 12 well plates (VWR) in NBM supplemented as above. The next day the culture media was changed to NBM supplemented with 2% B27, 1% penicillin-streptomycin and 1% L-Glutamine. Neurons were maintained at 37 °C with 5% CO_2_, and ~50% of the media was changed every 3–4 days. Neurons were cultured in control conditions (as above), with 0.1% DMSO vehicle control, or with 20 μM PD150606 (Tocris) dissolved in DMSO.

### Molecular Biology

Protein sample preparation, subcellular fractionation, and Western blotting (WB) was performed as previously described, and all protein samples were prepared in solutions containing broad-spectrum protease inhibitors^6^. Blocking and antibody incubations of WB PVDF membranes were performed with 5% skim milk powder + 0.1% Tween-20 in 1 × PBS for 1 h at room temperature or overnight at 4 °C (menin C-terminal epitope [Bethyl Laboratories, A300-105A, 1:2000]; menin N-terminal epitope [Santa Cruz Biotechnology, sc-374371, 1:2000]; histone H3 [Millipore, 06-599, 1:2000]; β-tubulin [Sigma-Aldrich, T0198, 1:2000]; synaptophysin [AbCam, ab52636, 1:2000]; IRDye-800CW conjugated α-mouse or α-rabbit IgG [Li-Cor biosciences, 925-32210, 925-32211, 1:5000]). WB membranes were visualized with a Li-Cor Odyssey infra-red imager, and bands were quantified using Odyssey v3.0 software. Li-Cor Odyssey infra-red imager settings are shown in Table S10. RNA samples were obtained from DIV 7 mouse hippocampal cultures with the RNeasy Plus Micro Kit (Qiagen). cDNA was synthesized with the QuantiTect Reverse Transcription Kit (Qiagen) and purified with a NucleoSpin PCR Clean-up column (Macherey-Nagel). qPCR was performed with SYBRgreen (Qiagen) and primers directed to a region of 80–120 bases. Kits were used according to manufacturers’ instructions. Intron spanning gene specific primers are shown in Table S11. Efficiency values for qPCR primers ranged between 85–110% (R^2^ = 0.97–1.00). Negative controls and validations were as previously described^5^. Relative gene expression (normalized to β-actin and β-tubulin reference genes), and statistical significance was determined using REST-2009^68^.

### Lentivirus Production and Transduction of Neuronal Cultures

Small hairpin (sh)RNA-encoding constructs were designed against *MEN1* or a non-target control (NTC) sequence and cloned into pLL3.7 (Addgene). *MEN1* shRNA sequence: CCGGTACCACTGTCGCAACCGAAATCTCGAGATTTCGGTTGCGACAGTGGTATTTTTG. NTC shRNA sequence: CGCGATAGCGCTAATAATTTCTCGAGAAATTATTAGCGCTATCGCGCTTTTTG. HEK-293 cells were cultured in DMEM supplemented with 10% FBS and 1% penicillin-streptomycin (GIBCO) and maintained at 37 °C with 5% CO_2_. HEK-293 cells were transfected with a mixture of pLL3.7 containing the *MEN1* or NTC shRNA sequence, along with psPAX2 and pMD2.G (Addgene) using HEPES/CaPO_4_ precipitation. After 4 h the media was replaced with 14 mL fresh media. After 24 h, lentivirus-containing media was harvested and replaced with 14 mL fresh media. Harvested media was centrifuged at 5,000 rpm for 5 m, filtered and stored at 4 °C, for a total of three harvests. The harvested media was ultracentrifuged for 2 h at 50,000× g using a SW 28 Ti rotor (Beckman). Lentivirus pellets were resuspended in 1 × PBS and stored in aliquots at −80 °C. Viral titers were determined using serial dilutions in HEK-293 cells. Transduction efficiency was estimated by visualization of GFP fluorescence after 24 h indicating viral titers of ~10^9^ IU/mL. Mouse hippocampal cultures were transduced with NTC shRNA- or *MEN1* shRNA-encoding lentivirus at DIV 1 by spinoculation (2 m at 2,000 rpm) using a multiplicity of infection of ~0.2, and the media was changed after 24 h. GFP fluorescence was observed after ~24–48 hours, and transduction efficiency of mouse hippocampal neurons was estimated to be ~60–80%. *MEN1* knockdown was confirmed by qPCR and ICC.

### Immunocytochemistry and Microscopy

Hippocampal cultures were fixed at DIV 3–14 for 30 m with 4% paraformaldehyde and 0.2% picric acid (Sigma Aldrich) in 1 × PBS, and permeabilized for 1 h with incubation medium (IM) containing 0.5% Triton and 10% goat serum in 1 × PBS. Primary antibodies (menin C-terminal epitope [Bethyl Laboratories, A300–105A]; menin N-terminal epitope [Santa Cruz Biotechnology, sc-374371]; neurofilament [Novus Biologicals, NB300-222]; NeuN [EMD Millipore, MAB377]; GFAP [AbCam, 16997]; synaptotagmin [EMD Millipore, MAB5200]; PSD-95 [Antibodies Incorporated, 75-028]; nAChR α5 [AbCam, ab41173-100]; GFP [Invitrogen, A11120, A11122]) were used at 1:500 in IM for 1 h. Secondary antibodies (Alexa Fluor 488, 546, or 633 conjugated goat α-rabbit, α-mouse or α-chicken [Invitrogen]) were used at 1:100 in IM for 1 h. α7-nAChR were labeled with Alexa Fluor 555 conjugated α-Bungarotoxin (Invitrogen, B35451) at 2 µg/mL in IM for 1 h. Three 15 m washes in 1x PBS were performed after each incubation, and all incubations were performed at room temperature. Cells were mounted using ProLong Gold antifade reagent with DAPI (Invitrogen). Confocal Z-stack imaging was performed as previously described^6^, using an A1R MP microscope under a CFI Plan Fluor 20x/0.75 MI objective, and images were acquired with NIS Elements v4.13.00 software (Nikon). Fluorophores were excited with 402, 488, 561 and 651 laser wavelengths and emissions collected through 450/50, 525/50 and 700/75 filter cubes. Super resolution imaging was performed with fluorescence super-resolution imaging modality Conical Diffraction Microscopy (CoDiM), which consists of a beam-shaping unit and a reconstruction processing algorithm^69^. The CoDiM100 module (BioAxial) was coupled to a C2 confocal microscope under an Apo TIRF 60x Oil DIC N2 objective (Nikon). Fluorophores were excited with 488, 561 and 640 laser wavelengths and emissions collected through 525/50, 598/44 and 710/50 filter cubes. The fibered output of the laser source of the microscope was diverted through the CoDiM100 beam-shaper module. The laser beam was thereafter coupled to the input port of the scanning head of the microscope. Four differently shaped intensity patterns were utilized to illuminate the sample. Each pattern has a topology which gains access to independent structural information of the sample. The fluorescent light was collected on an Orca Flash 4 V2 sCMOS camera (Hamamatsu Photonics). Each scanning point in the sample plane was hence interrogated four times by the excitation light, generating four different micro images. To obtain the super-resolved image the micro images were then processed by a dedicated algorithm. Image processing, fluorescence intensity and puncta analyses were performed with ImageJ (NIH). Imaging parameters including field of view size, laser intensity and channel gain were kept the same amongst relevant samples. Microscope settings are shown in Table S12. Images were collected from ≥2 samples prepared from independent culture sessions.

### Electrophysiology

Whole-cell patch-clamp recordings of spontaneous synaptic currents were made at a holding potential of −70 mV in the presence of 0.5 µM tetrodotoxin from pyramidal neurons in untreated control, NTC shRNA- or *MEN1* shRNA-encoding lentivirus transduced mouse hippocampal cultures (GFP+ neurons were selected from lentivirus-transduced cultures; transduction efficiency ~60–80%) at DIV 10–14, as previously described for rat cortical cultures^70^. Nicotine (Sigma Aldrich) was dissolved into the external recording solution (10 µM) and applied using pressure application through a microelectrode (tip opening ~1–5 µm; 250 ms pulse, 10 PSI). Nicotine-induced facilitation was determined by analyzing the frequency and amplitude of spontaneous synaptic events that occurred during a 10 s interval before and after the nicotine pulse. Vehicle application did not alter mEPSC amplitude or frequency (Fig. S7). Data analysis was performed with MiniAnalysis v6.0.7 (Synaptosoft Inc), traces were visually screened and synaptic events exhibiting appropriate waveforms with a monophasic rise time to peak were selected for detection and measurement by the software to determine the amplitude and frequency of mEPSCs.

### Experimental Design and Statistical Analysis

Data sets were derived from ≥2 independent experiments, using samples from ≥2 independent cell culture preparations or tissue collected from ≥3 animals, to ensure results were reliable and replicable. Minimum sample sizes for quantitative data were determined using the resource equation method. Data analyses were performed blinded by acquisition file number. Statistical analyses were performed using SPSS Statistics v22 for Windows. The distribution of data was analyzed with the Shapiro-Wilk test of normality, and parametric (*P* > 0.05) or non-parametric (*P* < 0.05) statistical tests were used as appropriate. Differences in fluorescence intensity for ICC were assessed with the Mann-Whitney U test or Student’s independent samples t-test (2-sided). Incidence of colocalization for super resolution imaging was assessed with the Kruskal-Wallis test, Student’s independent samples t-test or Mann-Whitney U test (2-sided). The incidence of nicotine-induced presynaptic facilitation was assessed with a Chi-squared test. Amplitude and frequency of synaptic events were assessed with univariate ANOVA with Tukey’s HSD *post hoc* test (Levene’s test *P* > 0.05), and nicotine-induced facilitation was assessed with Student’s paired samples t-tests (2-sided). Significant differences in relative gene expression were determined via pair wise fixed reallocation randomization test using REST-2009^68^.

## Electronic supplementary material

supplemental information
