# Supplementary material for: Tumor suppressor menin is required for subunit-specific nAChR α5 transcription and nAChR-dependent presynaptic facilitation in cultured mouse hippocampal neurons
Source: Sci Rep. 2017 May 11;7:1768. doi: 10.1038/s41598-017-01825-x (PMC5432004; doi:10.1038/s41598-017-01825-x)
Supplement: Supplementary file 1 — supplemental information [file 41598_2017_1825_MOESM1_ESM.pdf]

SUPPLEMENTARY INFORMATION FOR

Tumor suppressor menin is required for subunit-specific nAChR α5 transcription and nAChR-dependent presynaptic facilitation in cultured mouse hippocampal neurons

AUTHORS

Angela M. Getz, Fenglian Xu, Frank Visser, Roger Persson, & Naweed I. Syed

This File Contains:

Supplementary Data:

Tables S1 to S9

Figures S1 to S7

Supplementary Experimental Procedures:

Tables S10 to S12

Supplementary References

SUPPLEMENTARY DATA

Supplementary Table S1. Related to Figure 2. ICC fluorescence of menin in hippocampal neuron cultures.

Treatment	Menin (N) Nuclear Fluorescence ± SEM (AU)	*P* [1]	n [3]
0.1% DMSO	87.30 ± 2.72	0.646	67
20 μM PD150606	85.35 ± 2.80		59
Treatment	Menin (C) Neurite Fluorescence ± SEM (AU)	*P* [2]	n [4]
0.1% DMSO	55.72 ± 3.19	<0.001	27
20 μM PD150606	38.86 ± 3.13		27

1. Mann-Whitney U test (2-tailed)
2. Independent samples t-test (2-tailed)
3. n refers to the analysis of menin N-terminal epitope fluorescence intensity within neuronal nuclei (delineated by DAPI), each neuron in the image was analyzed, ROIs derived from 9 separate images from 3 independent experiments
4. n refers to the analysis of menin C-terminal epitope fluorescence intensity along a 25 μM length of neurite (delineated by the neurofilament immunolabel), 3 neurites from 3 different neurons were analyzed per image, ROIs derived from 9 separate images from 3 independent experiments

Supplementary Table S2. Related to Figure 3. Incidence of C-menin colocalization in super-resolved ICC images.

ICC Label	Mean Number of Puncta ± SEM	% Colocalization with C-menin ± SEM	*P* [1]	n [2]
Synaptotagmin	43.89 ± 5.72	61.02 ± 7.53	<0.001	9
PSD-95	46.00 ± 3.55	29.11 ± 2.87	<0.001	10
α-BTX	45.79 ± 4.19	93.40 ± 2.01	-	19
Menin (C)	41.94 ± 3.25	-	-	19

1. Kruskal-Wallis test (2-tailed), statistical significance (*P*) relative to C-menin and α-BTX colocalization
2. n refers to ≥9 separate images from ≥2 independent experiments

Supplementary Table S3. Related to Figure 4. Relative gene expression in lentivirus-transduced hippocampal neuron cultures.

Treatment	qPCR Target	Fold-Change Expression [1]	SEM	*P* [2,3]
Untreated Control<br><br>n=6	*MEN1*	1	0.847 - 1.181	-
	nAChR α 2	1	0.803 - 1.245	-
	nAChR α 3	1	0.610 - 1.641	-
	nAChR α 4	1	0.863 - 1.159	-
	nAChR α 5	1	0.722 - 1.386	-
	nAChR α 6	ND [4]	-	-
	nAChR α 7	1	0.781 - 1.280	-
	nAChR β 2	1	0.851 - 1.175	-
	nAChR β 3	ND	-	-
	nAChR β 4	1	0.377 - 2.650	-
	GluR1	1	0.908 - 1.101	-
	NR2A	1	0.847 - 1.181	-
	Synaptophysin	1	0.863 - 1.158	-
NTC shRNA<br><br>n=6	*MEN1*	0.812	0.649 - 1.179	0.195
	nAChR α 2	1.212	0.925 - 1.609	0.148
	nAChR α 3	1.181	0.683 - 1.907	0.394
	nAChR α 4	1.213	0.955 - 1.463	0.059
	nAChR α 5	1.315	0.935 - 1.831	0.098
	nAChR α 6	ND	-	-
	nAChR α 7	1.002	0.713 - 1.365	0.982
	nAChR β 2	1.564	1.117 - 2.340	0.014
	nAChR β 3	ND	-	-
	nAChR β 4	0.795	0.324 - 1.783	0.522
	GluR1	1.203	1.010 - 1.496	0.061
	NR2A	0.651	0.449 - 0.971	0.019
	Synaptophysin	1.174	0.929 - 1.551	0.165
MEN1 shRNA<br><br>n=6	*MEN1*	0.593	0.430 - 0.933	0.009
	nAChR α 2	2.964	2.200 - 3.910	0.001
	nAChR α 3	2.983	1.661 - 5.317	0.001
	nAChR α 4	1.569	1.059 - 2.198	0.019
	nAChR α 5	0.343	0.256 - 0.446	0.002
	nAChR α 6	ND	-	-
	nAChR α 7	2.196	1.553 - 3.183	0.001
	nAChR β 2	3.728	2.762 - 5.242	0.001
	nAChR β 3	ND	-	-
	nAChR β 4	1.392	0.515 - 3.080	0.383
	GluR1	2.658	2.184 - 3.274	0.001
	NR2A	3.729	2.476 - 6.935	<0.001
	Synaptophysin	2.727	2.220 - 3.325	0.001

1. Fold-change expression relative to untreated control
2. Pair wise fixed reallocation randomization test (REST-2009)[1]
3. Statistical significance (*P*) relative to untreated control
4. ND indicates the transcript was not detected in the qPCR reaction

Supplementary Table S4. Related to Figure 5. Mean somal ICC fluorescence values of menin and nAChRs in lentivirus-transduced hippocampal neuron cultures.

Treatment	DIV	Menin (C) ± SEM (AU)	*P* [1]	n [2]	Menin (N) ± SEM (AU)	*P*	n
NTC shRNA GFP+	3	59.64 ± 5.83	0.457	28	71.66 ± 4.42	0.972	36
NTC shRNA GFP-		55.31 ± 4.15		42	71.85 ± 4.63		27
NTC shRNA GFP+	7	76.03 ± 4.28	0.420	33	71.76 ± 3.30	0.981	49
NTC shRNA GFP-		72.28 ± 3.85		38	71.95 ± 3.91		33
NTC shRNA GFP+	10	56.91 ± 3.24	0.751	29	96.67 ± 3.50	0.517	32
NTC shRNA GFP-		58.20 ± 2.39		34	100.68 ± 5.74		30
NTC shRNA GFP+	14	52.93 ± 3.49	0.648	25	98.58 ± 4.54	0.652	43
NTC shRNA GFP-		52.48 ± 2.26		30	97.59 ± 4.48		46
Treatment	DIV	nAChR α5 ± SEM (AU)	*P*	n	α-BTX ± SEM (AU)	*P*	n
NTC shRNA GFP+	3	49.47 ± 3.77	0.417	27	72.67 ± 4.54	0.588	45
NTC shRNA GFP-		50.75 ± 6.70		18	69.13 ± 4.61		52
NTC shRNA GFP+	7	46.51 ± 2.31	0.634	24	103.29 ± 5.72	0.521	53
NTC shRNA GFP-		48.14 ± 2.41		30	99.22 ± 4.81		54
NTC shRNA GFP+	10	50.27 ± 2.07	0.898	23	117.11 ± 5.00	0.978	48
NTC shRNA GFP-		49.05 ± 2.11		24	114.93 ± 5.35		51
NTC shRNA GFP+	14	46.84 ± 2.76	0.689	30	50.27 ± 2.07	0.682	23
NTC shRNA GFP-		46.70 ± 2.27		24	49.05 ± 2.11		24
Treatment	DIV	Menin (C) ± SEM (AU)	*P*	n	Menin (N) ± SEM (AU)	*P*	n
MEN1 shRNA GFP+	3	40.12 ± 2.42	<0.001	28	60.07 ± 2.88	0.004	54
MEN1 shRNA GFP-		64.16 ± 3.34		37	74.58 ± 4.07		45
MEN1 shRNA GFP+	7	43.85 ± 1.69	<0.001	50	65.38 ± 2.72	0.001	58
MEN1 shRNA GFP-		60.56 ± 3.44		26	79.71 ± 3.40		44
MEN1 shRNA GFP+	10	53.02 ± 3.32	0.003	24	83.77 ± 4.77	0.602	47
MEN1 shRNA GFP-		70.88 ± 4.89		17	79.41 ± 5.65		27
MEN1 shRNA GFP+	14	43.52 ± 3.23	0.006	14	56.86 ± 3.96	0.473	35
MEN1 shRNA GFP-		62.60 ± 5.42		13	55.31 ± 5.53		27
Treatment	DIV	nAChR α5 ± SEM (AU)	*P*	n	α-BTX ± SEM (AU)	*P*	n
MEN1 shRNA GFP+	3	40.21 ± 3.76	0.984	24	75.67 ± 4.26	0.024	54
MEN1 shRNA GFP-		42.20 ± 4.32		25	90.06 ± 4.58		61
MEN1 shRNA GFP+	7	41.10 ± 2.19	<0.001	36	111.46 ± 3.17	0.456	86
MEN1 shRNA GFP-		57.63 ± 2.63		29	115.31 ± 4.02		51
MEN1 shRNA GFP+	10	42.35 ± 3.13	0.001	18	96.56 ± 4.14	0.682	58
MEN1 shRNA GFP-		59.91 ± 3.32		22	99.23 ± 4.85		36
MEN1 shRNA GFP+	14	41.37 ± 2.72	<0.001	12	97.30 ± 10.14	0.186	14
MEN1 shRNA GFP-		58.96 ± 2.90		23	115.60 ± 8.71		13

1. Independent samples t-test or Mann-Whitney U test (2-tailed)
2. n refers to the analysis of fluorescence intensity within neuronal soma, ROIs derived from ≥4 separate images from ≥2 independent experiments, GFP+ and GFP- neurons were analyzed from the same images

Supplementary Table S5. Related to Figure 6. Mean neurite ICC fluorescence values of menin and nAChRs in lentivirus-transduced and calpain inhibitor-treated hippocampal neuron cultures.

Treatment	Menin (C) Neurite Fluorescence ± SEM (AU)	*P* [1]	n [2]
NTC shRNA	30.59 ± 1.07	<0.001	51
MEN1 shRNA	13.70 ± 0.93		52
Treatment	α-BTX Neurite Fluorescence ± SEM (AU)	*P*	n
NTC shRNA	26.14 ± 1.69	0.038	51
MEN1 shRNA	20.08 ± 1.05		52
0.1% DMSO	29.03 ± 1.28	<0.001	36
20 μM PD150606	21.40 ± 0.85		36

1. Mann-Whitney U test (2-tailed)
2. n refers to the analysis of fluorescence intensity values along a 25 μM length of neurite (delineated by GFP fluorescence or the neurofilament immunolabel), 2-3 neurites from different neurons were analyzed per image, ROIs derived from ≥12 separate images from ≥2 independent experiments

Supplementary Table S6. Related to Figure 7. Incidence of puncta colocalization in super-resolved ICC images from NTC shRNA- and *MEN1* shRNA-transduced hippocampal neuron cultures.

shRNA Treatment	Mean Number of Puncta ± SEM								
	C-menin	*P* [1]	n [2]	α-BTX	*P*	n	SYT	*P*	n
NTC	57.37 ± 1.84	<0.001	27	57.62 ± 2.62	0.002	26	40.65 ± 2.04	0.790	31
MEN1	44.63 ± 2.79		24	46.08 ± 2.30		26	40.00 ± 1.30		30
shRNA Treatment	% Colocalization ± SEM								
	SYT - C-menin	*P*	n	SYT – α-BTX	*P*	n	α-BTX - C-menin	*P*	n
NTC	55.63 ± 2.50	<0.001	16	68.81 ± 3.03	0.033	15	96.17 ± 1.25	0.430	11
MEN1	36.23 ± 1.97		14	59.01 ± 3.14		16	97.24 ± 1.24		10

1. Independent samples t-test or Mann-Whitney U test (2-tailed)
2. n refers to ≥10 separate images from ≥2 independent experiments

Supplementary Table S7. Related to Figure S5. mEPSC values in lentivirus-transduced hippocampal neuron cultures (all neuron samples).

Treatment	10 µM Nicotine	Mean Amplitude (pA) [1]	SEM (pA)	*P* [2]	*P* [3]	n
Untreated Control	Pre	33.09	2.88	(F=0.405; *P*=0.844)	0.800	19
	Post	32.55	2.48	1.000		
NTC shRNA	Pre	37.69	2.86	0.912	0.259	15
	Post	35.95	2.60	0.988		
MEN1 shRNA	Pre	36.33	3.99	0.976	0.717	17
	Post	35.92	3.89	0.987		
Treatment	10 µM Nicotine	Mean Frequency (Hz) [1]	SEM (Hz)	*P*	*P*	n
Untreated Control	Pre	3.60	0.58	(F=1.373; *P*=0.241)	<0.001	19
	Post	4.87	0.67	0.494		
NTC shRNA	Pre	3.66	0.50	1.000	<0.001	15
	Post	4.86	0.55	0.571		
MEN1 shRNA	Pre	3.74	0.37	1.000	0.628	17
	Post	3.61	0.43	1.000		

1. Data analysis was performed using MiniAnalysis software (Synaptosoft Inc), which detects and measures the characteristics of mEPSCs when a ROI is selected. Traces were visually screened and synaptic events exhibiting a waveform appropriate for mEPSCs with a monophasic rise time to peak were selected for analyses. The total mean mEPSC rise and decay times was 5.70 ± 0.17 and 13.37 ± 0.59, respectively, and did not vary amongst treatment groups or after nicotine application ($P \geq 0.231$, one-way ANOVA; $P \geq 0.119$, Kruskal-Wallis test). The total mean baseline noise was ± 5.81 pA.
2. One-way ANOVA, statistical significance (*P*) relative to untreated control, pre-nicotine
3. Paired samples t-test (2-tailed)

Supplementary Table S8. Related to Figure S5 and Figure 8. Incidence of nicotine-induced facilitation in lentivirus-transduced hippocampal neuron cultures (all neuron samples).

Treatment	Relative mEPSC amplitude (Post/Pre-nicotine) ± SEM	*P* [1]	
Untreated Control	1.03 ± 0.06	(F=0.517; *P*=0.600)	
NTC shRNA	0.96 ± 0.04	0.619	
MEN1 shRNA	1.00 ± 0.03	0.860	
Treatment	Relative mEPSC frequency (Post/Pre-nicotine) ± SEM	*P* [2]	
Untreated Control	1.49 ± 0.12	-	
NTC shRNA	1.45 ± 0.12	0.784	
MEN1 shRNA	0.98 ± 0.06	<0.001	
Treatment	Incidence of nicotine-induced presynaptic facilitation ± SEM (%) [3]	χ^2	*P* [4]
Untreated Control	n = 13/19; 68.42 ± 6.79	-	-
NTC shRNA	n = 11/15; 73.33 ± 12.47	0.097	0.755
MEN1 shRNA	n = 5/17; 29.41 ± 4.44	5.461	0.019

1. One-way ANOVA, statistical significance (*P*) relative to untreated control
2. Kruskal-Wallis test (2-tailed), statistical significance (*P*) relative to untreated control
3. Nicotine-induced presynaptic facilitation defined by the proportion of neurons exhibiting an increase in mEPSC frequency in response to nicotine application, with a relative mEPSC frequency (post-nicotine/pre-nicotine) of ≥ 1.00 ± SEM
4. Chi-squared test, statistical significance (*P*) relative to untreated control

Supplementary Table S9. Related to Figure 8. mEPSC values in lentivirus-transduced hippocampal neuron cultures (neuron samples exhibiting nicotine-induced presynaptic facilitation only).

Treatment	10 µM Nicotine	Mean Amplitude (pA)	SEM (pA)	*P* [1]	*P* [2]	n
Untreated Control	Pre	29.83	2.81	(F=0.857; *P*=0.516)	0.800	13
	Post	30.49	3.23	1.000		
NTC shRNA	Pre	37.82	3.60	0.543	0.130	11
	Post	34.99	3.36	0.882		
MEN1 shRNA	Pre	36.28	5.70	0.893	0.275	5
	Post	34.73	6.05	0.965		
Treatment	10 µM Nicotine	Mean Frequency (Hz)	SEM (Hz)	*P*	*P*	n
Untreated Control	Pre	3.20	0.59	(F=1.616; *P*=0.172)	<0.001	13
	Post	4.99	0.78	0.334		
NTC shRNA	Pre	3.37	0.54	1.000	<0.001	11
	Post	4.99	0.62	0.383		
MEN1 shRNA	Pre	3.08	0.70	1.000	0.130	5
	Post	4.14	1.16	0.967		

1. One-way ANOVA, statistical significance relative to untreated control, pre-nicotine
2. Paired samples t-test (2-tailed)

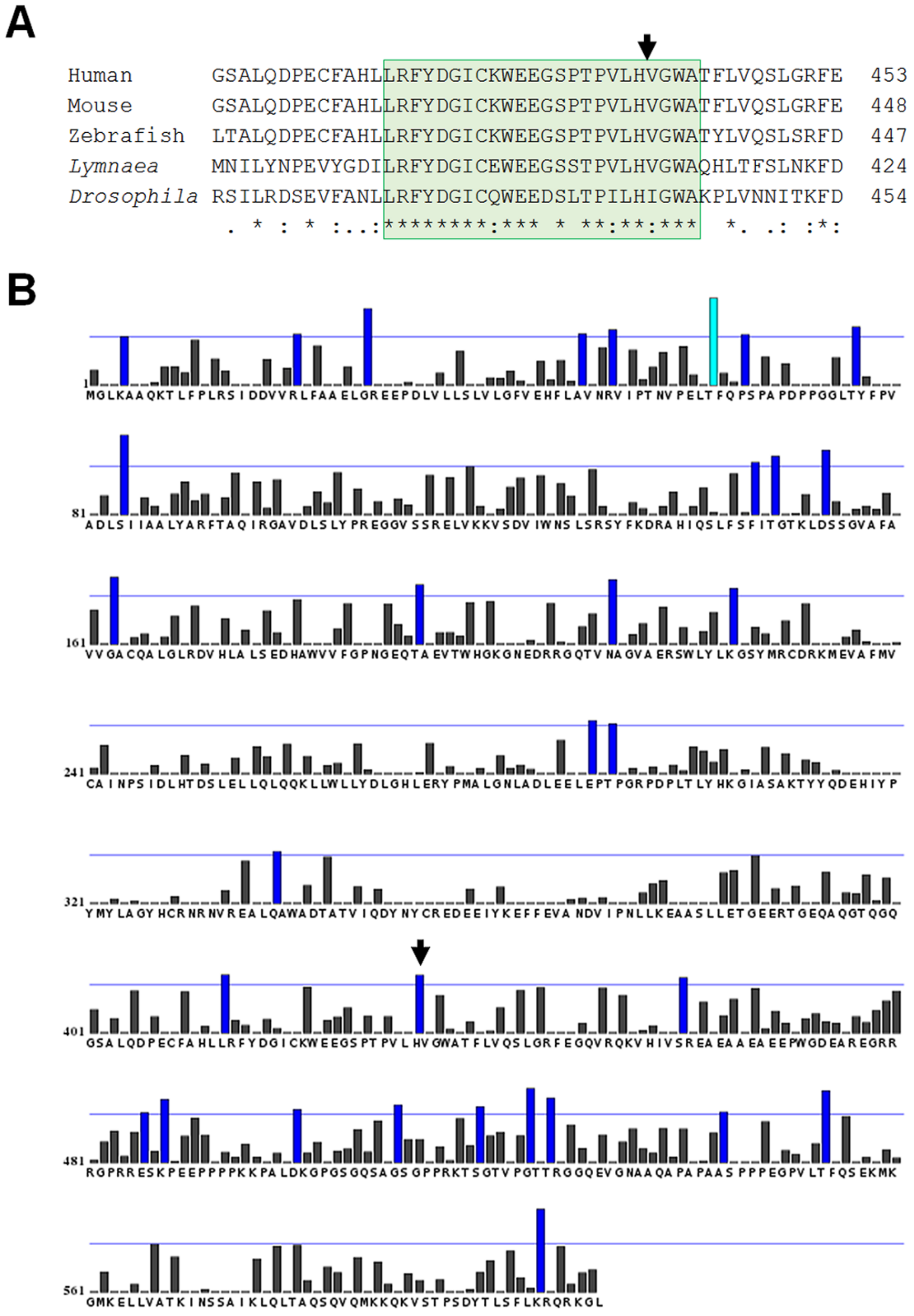

<u>Supplementary Figure S1. Related to Figure 1. The conserved menin ROI and calpain cleavage site.</u>

(**A**). Sequence alignment of the conserved ROI of menin (shaded green) from vertebrate species human, mouse and zebrafish, and invertebrate species *Lymnaea* and *Drosophila*. Arrow indicates an evolutionarily conserved calpain protease consensus sequence. (**B**). Multiple kernel learning prediction[2] identifies the presumptive calpain cleavage site in the conserved ROI of mouse menin (arrow). Blue line indicates threshold for significant prediction scores, blue bars indicates predicted calpain cleavage sites.

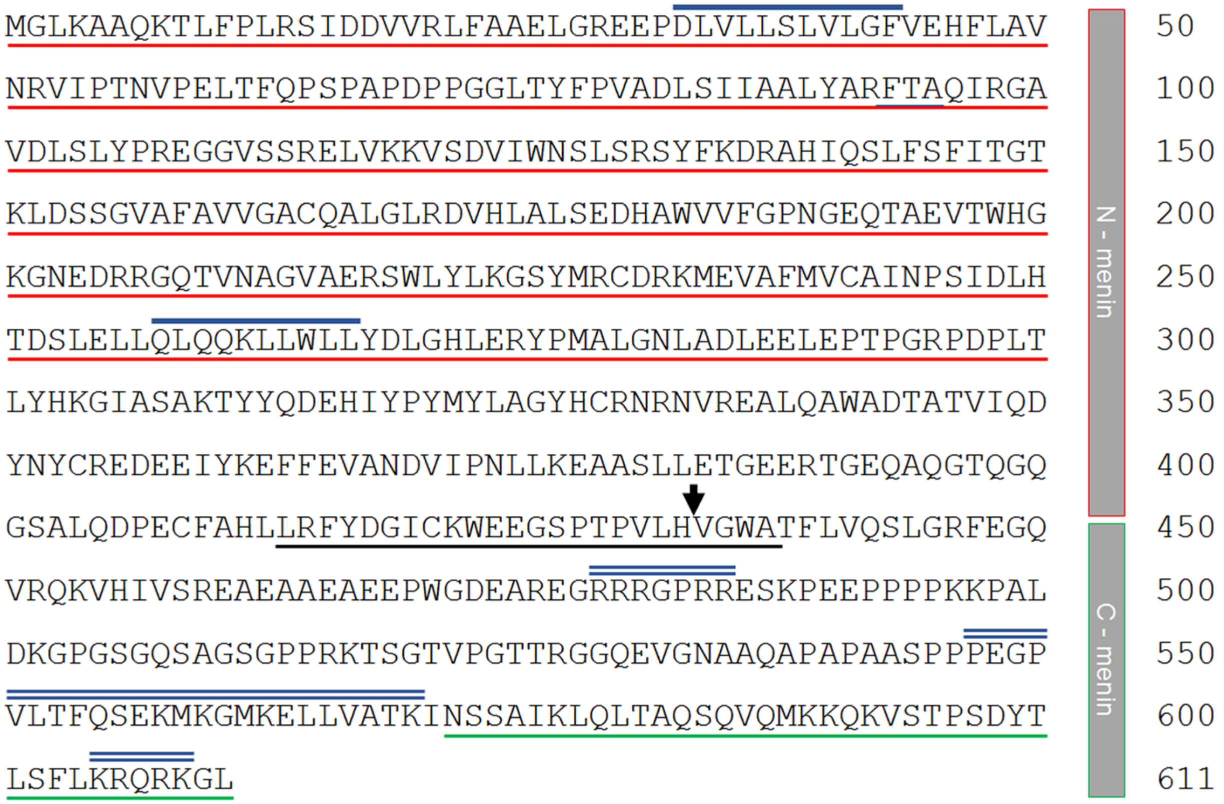

<u>Supplementary Figure S2. Related to Figure 1. The N- and C-terminal epitopes recognized by menin antibodies.</u>

Mouse menin sequence (accession no. AF016398.1). The N-terminal epitope recognized by the menin (N) antibody is underlined in red. The C-terminal epitope recognized by the menin (C) antibody is underlined in green. The conserved ROI is underlined in black, and the calpain cleavage site is indicated with an arrow. Locations of nuclear localization signals (blue, double bar) and nuclear exit signals (blue, single bar) are also shown.

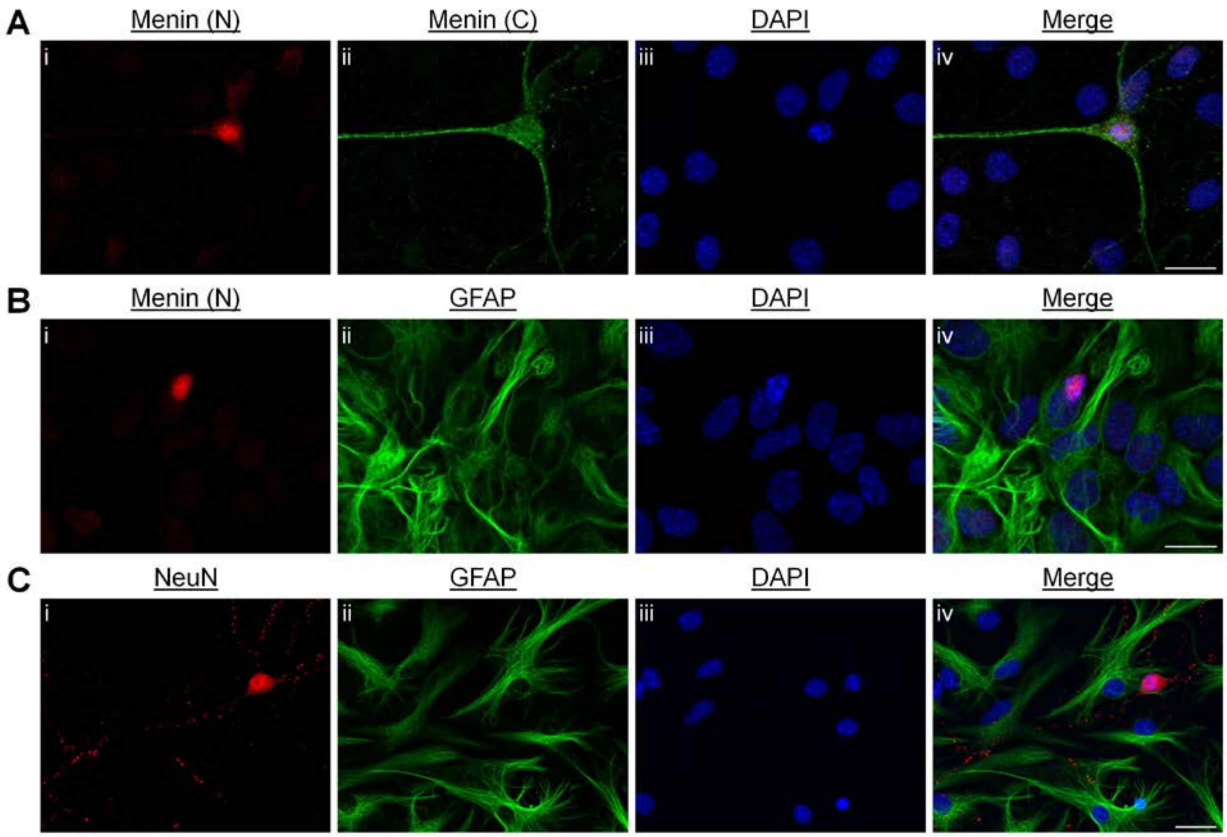

<u>Supplementary Figure S3. Related to Figures 1-2. Menin exhibits neuron-specific expression in hippocampal cultures.</u>

(**A-C**). ICC localization of menin and cell-type markers in hippocampal cultures at DIV 14 (representative images). (**A**). Cells labeled with N-terminal (**i**) and C-terminal (**ii**) epitope menin antibodies, and the nuclear stain DAPI (**iii**), (**iv**) shows merged channels (n=13 images, 4 independent samples). (**B**). Cells labeled with N-terminal menin (**i**) and GFAP (**ii**; glial marker) antibodies, and the nuclear stain DAPI (**iii**), (**iv**) shows merged channels (n=9 images, 3 independent samples). (**C**). Cells labeled with NeuN (**i**; neuronal marker) and GFAP (**ii**) antibodies, and the nuclear stain DAPI (**iii**), (**iv**) shows merged channels (n=7 images, 3 independent samples). α-menin signals were absent in glia. Scale bars, 20 μm.

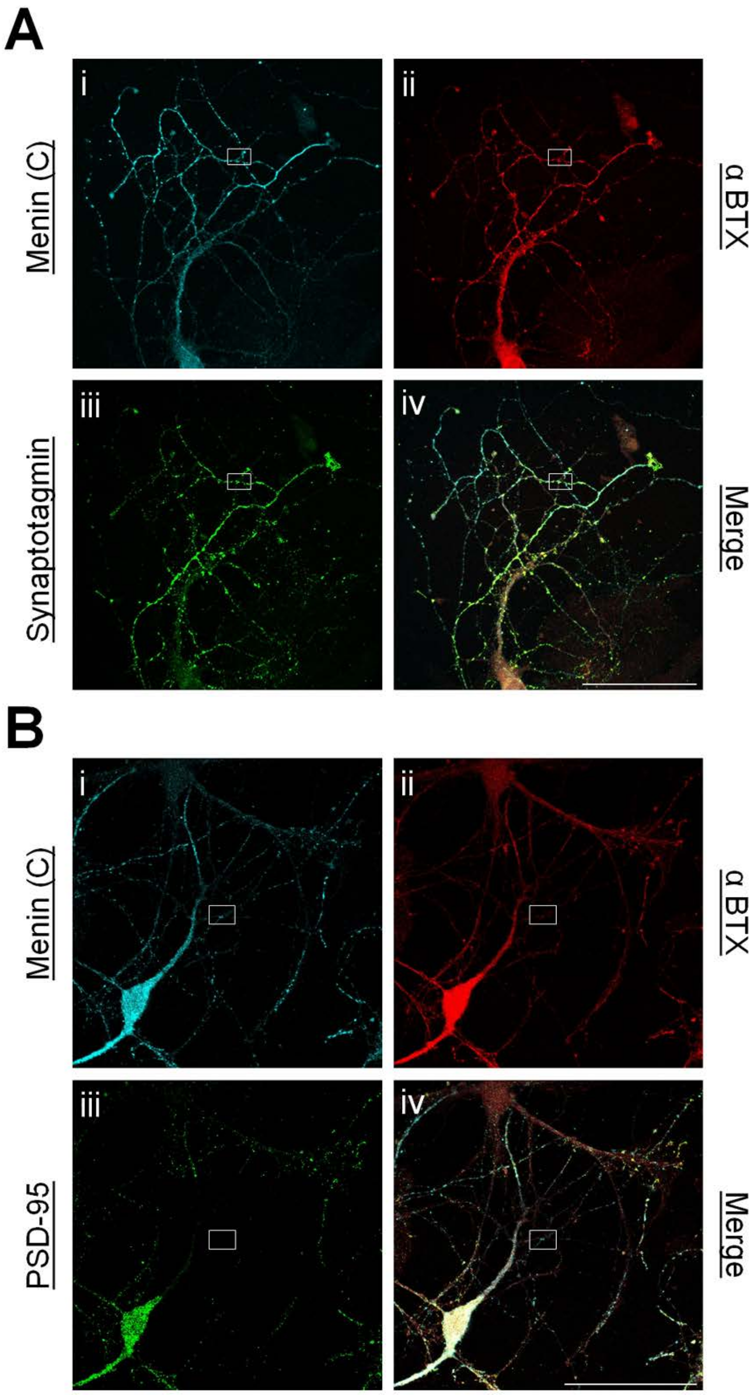

<u>Supplementary Figure S4. Related to Figure 3. Field of view images for super-resolution microscopy.</u>

(**A**). Mouse hippocampal neurons at DIV 7 labeled with α-BTX to detect α7 subunit-containing nAChR (**i**), a C-terminal epitope menin antibody (**ii**), and a synaptotagmin antibody to detect presynaptic sites (**iii**), (**iv**) shows merged channels. Boxed area is the synaptic ROI depicted with the super resolution image in Fig. 3A. (**B**). As in **A**, only labeled with a PSD-95 antibody to detect postsynaptic sites (**iii**). Boxed area is the synaptic ROI depicted in the super resolution image in Fig. 3B. Scale bars, 50 μm.

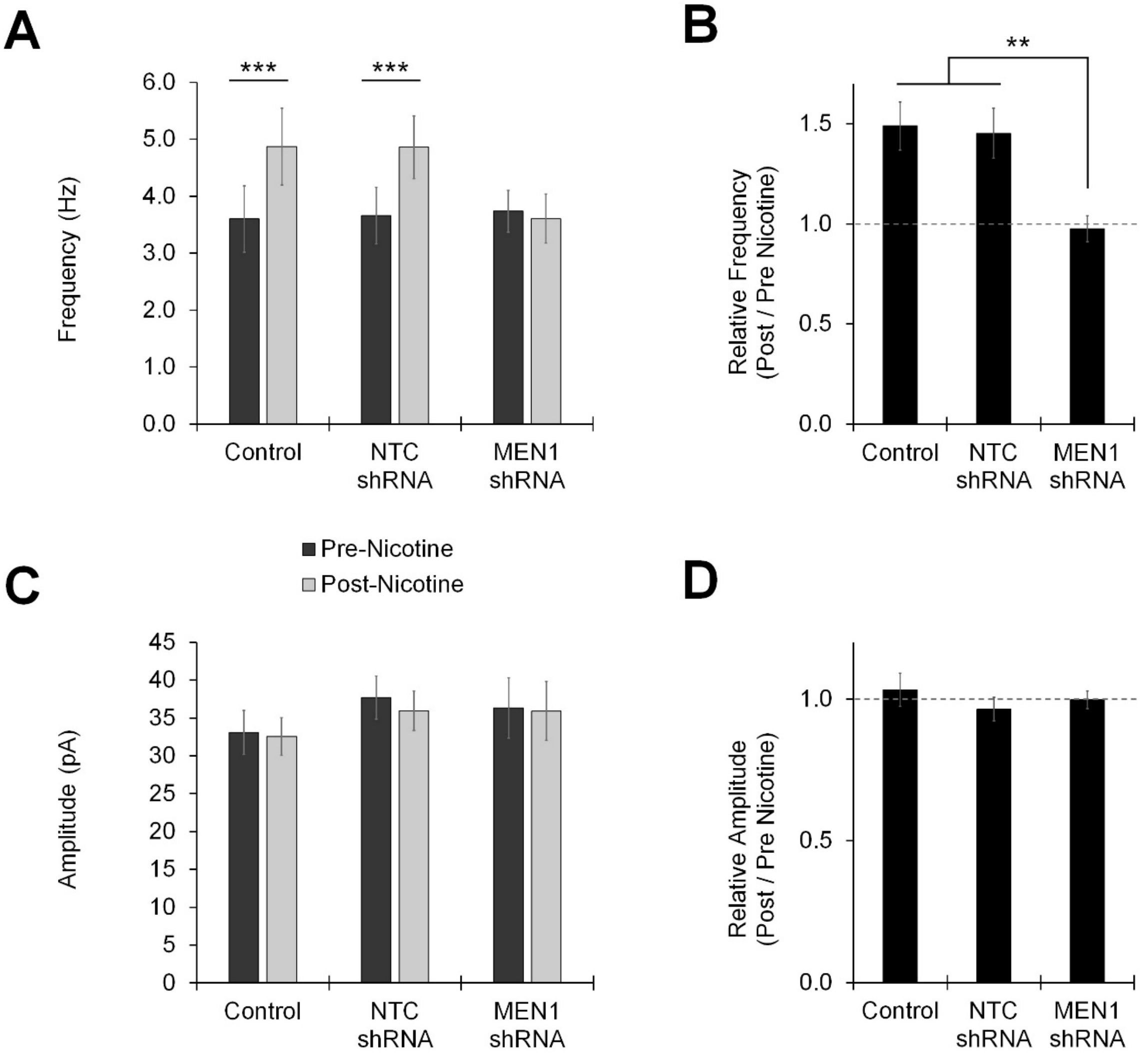

<u>Supplementary Figure S5. Related to Tables S7-S8 and Figure 8. mEPSC values in lentivirus-transduced hippocampal neuron cultures (all neuron samples).</u>

(**A**). Summary data, mean frequency of mEPSCs, before (Pre) and after (Post) the nicotine pulse recorded from neurons in untreated control (n=19), NTC shRNA (n=15; GFP+), and *MEN1* shRNA (n=17; GFP+) transduced cultures. ***, statistical significance (paired t-test), $P<0.001$. (**B**). Relative mEPSC frequency. **, statistical significance (Kruskal-Wallis test), $P\leq0.001$. (**C**). Summary data, mean amplitude of mEPSCs before (Pre) and after (Post) the nicotine pulse, as in (**A**). (**D**). Relative mEPSC amplitude. Dashed line represents a 1:1 ratio indicating no change.

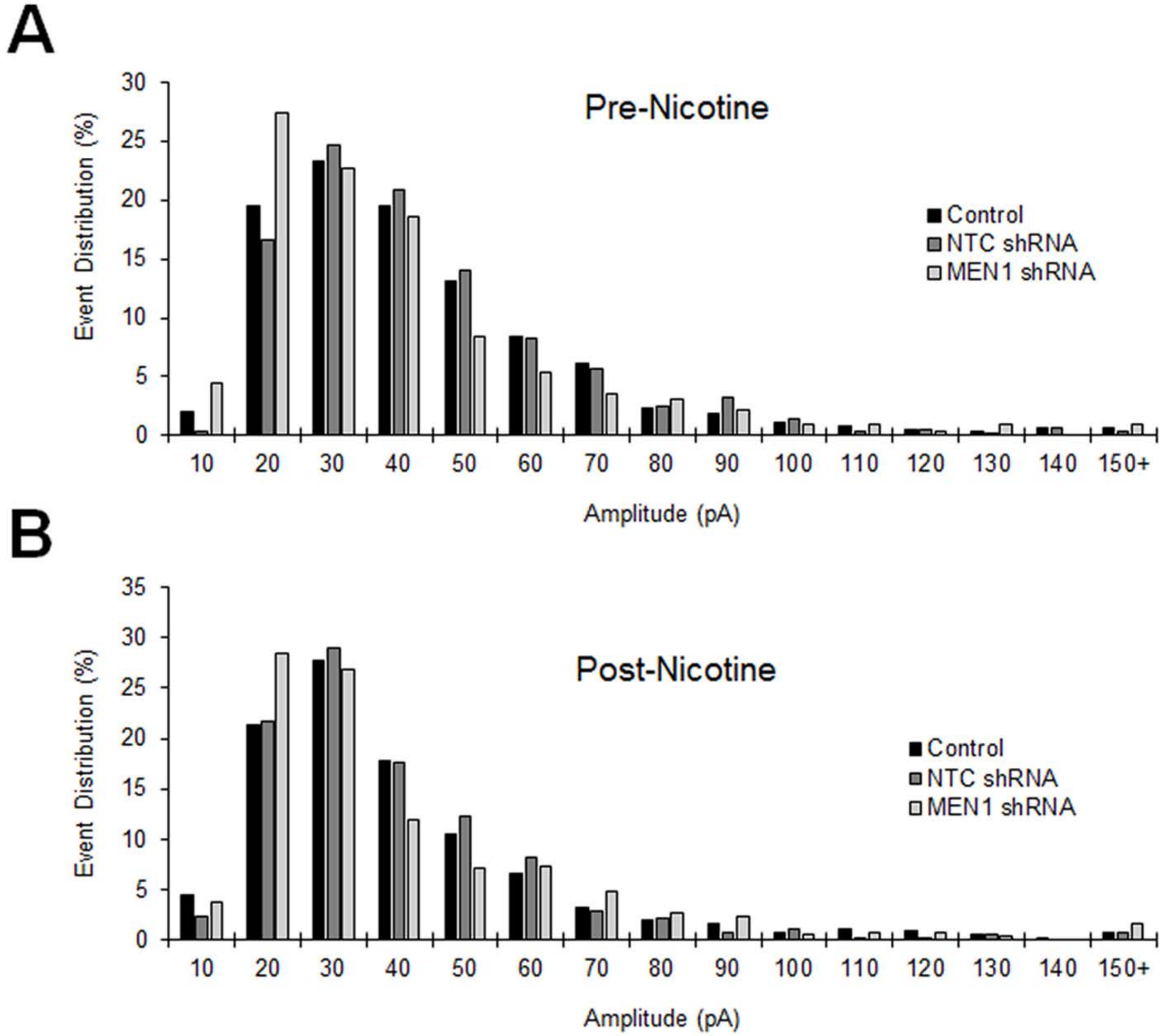

Supplementary Figure S6. Related to Figure S5 and Figure 8. Histogram of mEPSC amplitude distribution (all neuron samples).

Amplitude histogram, patch clamp recordings were made from mouse hippocampal neurons before (**A**) and after (**B**) the application of nicotine. Amplitudes of mEPSCs were binned in 10 pA increments, and are represented here as a percentage of the total number of spontaneous synaptic events that occurred during the 10s period analyzed before and after the application of nicotine.

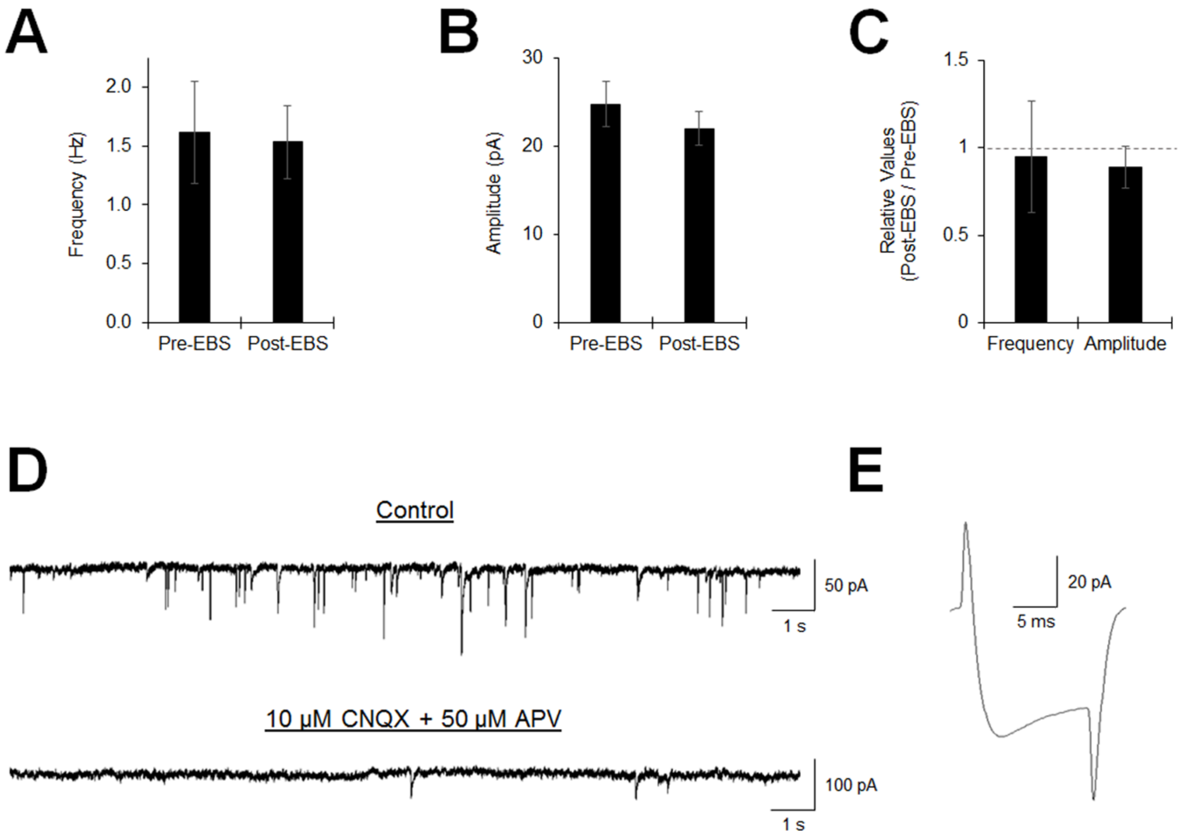

Supplementary Figure S7. Related to Figure 8. Control experiments for patch clamp recordings.

(**A-C**). Application of vehicle control (n=6; external recording solution [EBS], 250 ms pulse, 10 PSI) did not induce facilitation of mEPSC frequency (**A**; paired t-test, *P*=0.752) or amplitude (**B**; paired t-test, *P*=0.145). (**C**). Relative mEPSC frequency and amplitude. (**D**) mEPSCs were inhibited by glutamate receptor antagonists (n=4; 10 µM 6-cyano-7-nitroquinoxaline-2,3-dione [CNQX] + 50 µM DL-2-amino-5-phosphonovaleric acid [APV]), demonstrating that mEPSCs depict the vesicular release of glutamate. (**E**). No autaptic synaptic currents were observed after a depolarization step (n=6; -100 mV to +30 mV), indicating that mEPSCs represent interneuronal synaptic transmission.

SUPPLEMENTARY EXPERIMENTAL PROCEDURES

Supplementary Table S10. Li-Cor Odyssey infra-red imager settings.

Li-Cor	Intensity	Gain	Volts	Current
Channel 700 (NEB Pre-stained protein marker)	4	1	155.03	55.3
Channel 800 (IRDye-800 CW conjugated antibodies)	5	1	161.172	31.5

Supplementary Table S11. qPCR gene specific primers (intron spanning).

Target	Accession Number	5' Sequence	3' Sequence	Efficiency (%)
β Tubulin	NM_023279	AGTCAGCATGAGGGAGATCG	TGCAGGTCTGAGTCCCCTAC	99.42
β Actin	NM_007393	ACTGTCGAGTCGCGTCCA	GCAGCGATATCGTCATCCAT	101.44
MEN1	NM_008583	TCCAGTCCCTCTTCAGCTTC	CCAGATGGACATCTCTGAGACC	96.44
nAChR α 2	NM_144803	TGAGAAGAATCAAATGATGACCAC	GGAGGTGATGTTGCCAAAC	91.72
nAChR α 3	NM_145129	TCAAAGAAGCCATCCAAAGTG	CACCATGGCAACATACTTCC	85.10
nAChR α 4	NM_015730	CGGCCAGTAGCCAATATCTC	AGTCATGCCACTCCTGCTTC	90.92
nAChR α 5	NM_176844	TCTGGTTGAAGCAGGAATGG	GGATCCACAGAGAGTCTGAAGG	89.37
nAChR α 6	NM_021369	GTGGAGAATGTCTCCGATCC	CAGCCACAGATTGGTCTCC	103.38
nAChR α 7	NM_007390	CCTCTCAGTGGTCGTGACAG	AACCATGCACACCAATTCAG	99.83
nAChR β 2	NM_009602	ACTCTATGGCGCTGCTGTTC	GGATCCAAGAGATGCTCCAC	96.71
nAChR β 3	NM_173212	CCCTGTGTTGAATTCCAGTG	GGATTCCATCGTAATTTTTGG	91.00
nAChR β 4	NM_148944	AGCTCCTCCCAGCTCATCTC	AGCCAGATGCTGGTGGTC	87.76
GluR1	NM_008165	ACACAAAGGCCTGGAATGG	ATGGCTTGGAGAAGTCGATG	98.13
NR2A	NM_008170	TCATGATCCAGGAGGAGTTTG	ATCGGAAAGGCGGAGAATAG	95.35
SYP	NM_009305	GAGGGACCCTGTGACTTCAG	AGCCTGTCTCCTTGAACACG	92.11

Supplementary Table S12. Nikon A1R MP and C2 confocal fluorescent microscope settings.

Fluorescent microscope settings for data presented in:	Nikon A1R MP - Laser Wavelength	Laser Power	PMT HV
Fig. 1	402.7	2.6	90
	488.0	7.8	104
	561.7	10.0	103
Fig. 2	402.7	3.8	100
	488.0	7.8	95
	561.7	10.0	81
	639.9	9.5	98
Fig. 5	402.7	2.6	90
	488.0	7.8	68
	561.7	10	107
	639.9	9.5	104
Fig. 6Ai-v	402.7	2.6	90
	488.0	7.8	65
	561.7	10.0	104
	639.9	9.5	104
Fig. 6Bi-ii	402.7	3.8	100
	488.0	7.8	95
	639.9	9.5	98
Fig. 6Biv-v	402.7	7.2	95
	561.7	10.0	100
	639.9	9.5	80
Fluorescent microscope settings for data presented in:	Nikon C2 - Laser Wavelength	Laser Power	PMT HV
Fig. 3	488.0	10.0	90
	561.0	20.0	90
	640.0	20.0	90
Fig. 7	488.0	1.0	85
	561.0	5.0	90
	640.0	5.0	90

SUPPLEMENTARY REFERENCES

1 Pfaffl, M. W., Horgan, G. W. & Dempfle, L. Relative expression software tool (REST) for group-wise comparison and statistical analysis of relative expression results in real-time PCR. *Nucleic acids research* **30**, e36 (2002).

2 DuVerle, D. A., Ono, Y., Sorimachi, H. & Mamitsuka, H. Calpain cleavage prediction using multiple kernel learning. *PloS one* **6**, e19035, doi:10.1371/journal.pone.0019035 (2011).
